# High content-imaging drug synergy screening identifies specific senescence-related vulnerabilities of mesenchymal neuroblastomas

**DOI:** 10.1038/s41419-025-07933-1

**Published:** 2025-08-25

**Authors:** Sonja Herter, Marta Emperador, Kyriaki Smyrilli, Daniela Kocher, Simay Celikyürekli, Constantia Zeiser, Xenia Gerloff, Sina Kreth, Kai-Oliver Henrich, Kendra K. Maaß, Johanna Rettenmeier, Thomas G. P. Grünewald, Heike Peterziel, Frank Westermann, Anne Hamacher-Brady, Olaf Witt, Ina Oehme

**Affiliations:** 1 Hopp Children’s Cancer Center Heidelberg (KiTZ), 69120 Heidelberg, Germany; 2 National Center for Tumor Diseases (NCT), NCT Heidelberg, a partnership between DKFZ and Heidelberg University Hospital, Heidelberg, Germany; 3 Clinical Cooperation Unit Pediatric Oncology, German Cancer Research Center (DKFZ) and German Cancer Consortium (DKTK), 69120 Heidelberg, Germany; 4 Faculty of Biosciences, Heidelberg University, 69120 Heidelberg, Germany; 5 Medical Faculty, Heidelberg University, 69120 Heidelberg, Germany; 6 Faculty of Mathematics and Computer Science, Heidelberg University, 69120 Heidelberg, Germany; 7 Division of Translational Neuroblastoma Research, German Cancer Research Center (DKFZ), 69120 Heidelberg, Germany; 8 German Cancer Research Center (DKFZ), Division of Pediatric Neurooncology, Heidelberg, Germany; 9 Department of Pediatric Oncology, Hematology, Immunology and Pulmonology, Heidelberg University Hospital, Heidelberg, Germany; 10 Division of Translational Pediatric Sarcoma Research, German Cancer Research Center (DKFZ), German Cancer Consortium (DKTK), Heidelberg, Germany; 11 Institute of Pathology, Heidelberg University Hospital, Heidelberg, Germany; 12 Department of Molecular Microbiology and Immunology, Johns Hopkins University Bloomberg School of Public Health, Baltimore, MD USA

**Keywords:** Paediatric cancer, Senescence

## Abstract

Neuroblastomas encompass malignant cells with varying degrees of differentiation, ranging from adrenergic (adr) cells resembling the sympathoadrenal lineage to undifferentiated, stem-cell-like mesenchymal (mes) cancer cells. Relapsed neuroblastomas, which often have mesenchymal features, have a poor prognosis and respond less to anticancer therapies, necessitating the development of novel treatment strategies. To identify novel treatment options, we analyzed the sensitivity of 91 pediatric cell models, including patient-derived tumoroid cultures, to a drug library of 76 anti-cancer drugs at clinically relevant concentrations. This included 24 three-dimensionally cultured neuroblastoma cell lines representing the range of mesenchymal to adrenergic subtypes. High-throughput ATP-based luminescence measurements were compared to high-content confocal imaging. With machine learning-supported imaging analysis, we focused on changes in the lysosomal compartment as a marker for therapy-induced senescence and assessed the basal lysosomal levels in a subset of untreated mesenchymal versus adrenergic cells. We correlated these findings with pathway activity signatures based on bulk RNA and scRNAseq. Comprehensive image-based synergy screens with spheroid cultures validated the combined effects of selected drugs on proliferation and cytotoxicity. Mesenchymal models presented high basal lysosomal levels correlating with senescence-associated secretory phenotype (SASP) and sphingolipid metabolism pathways. Chemotherapy treatment further increased lysosome numbers, indicative of therapy-induced senescence. Furthermore, the mesenchymal subtypes correlated with MAPK activity and sensitivity to MAPK pathway inhibitors. Lysosomal and SASP signaling is druggable by inhibitors of lysosomal acid sphingomyelinase (SLMi) or senolytics, including BCL2-family inhibitors. Especially the sequential combination of MEK inhibitors (MEKi) with BCL2-family inhibitors was the most effective on relapsed neuroblastoma cell lines. Gene expression analysis of 223 patient samples, drug sensitivity profiling of five patient-derived fresh tissue cultures, and in vivo zebrafish embryo neuroblastoma xenograft models confirmed these findings. Inhibition of MAPK signaling in combination with BCL2-family inhibitors is a novel treatment option for patients suffering from relapsed neuroblastomas.

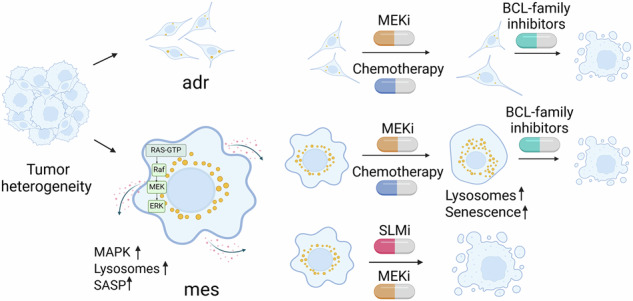

## Highlights

- Identification of specific vulnerabilities in neuroblastomas through comprehensive drug screening, aiming to address the poor prognosis and limited treatment options for relapsed neuroblastomas, focusing on mesenchymal and adrenergic subtypes.
- Complementation of high-throughput screening by high-content confocal imaging and imaging-based synergy screens, focusing on cell morphology and lysosomal response to drug treatment.
- Repurposing of clinically approved drugs identified MEK inhibitors and the combination of MEK inhibitors with BCL2-family inhibitors as a promising treatment option for relapsed neuroblastomas in vitro (cell lines), ex vivo (patient-derived organoid-like cultures), and in vivo (zebrafish embryo xenograft model).

## Introduction

Neuroblastoma is the most common extracranial solid tumor in children. It arises from the developing neural crest in early fetal development [1]. Neuroblastoma is a very heterogeneous disease; its outcome ranges from spontaneous regression to fatal outcomes in high-risk disease despite intensive therapy [2]. In addition to recurrent genetic lesions, epigenetic profiling revealed the existence of epigenetically regulated tumor subtypes, which include an adrenergic phenotype that is more differentiated and a more mesenchymal phenotype [3, 4]. The subtypes exhibit unique sets of lineage-specific super enhancers and core regulatory circuits. Expression of these gene sets characterizes them in a spectrum between mesenchymal and adrenergic [5]. The subtypes also diverge in clinical characteristics: mesenchymal-like cells are relatively enriched in relapsed tumors and are more resistant to the standard chemotherapy drugs cisplatin, doxorubicin and etoposide [3, 4]. However, comprehensive and systematic studies assessing the differences in sensitivity to a broader drug library, encompassing drugs matching molecular alterations, are missing.

During the malignant transformation of cells, proliferation and metabolic needs increase. Cellular stress response pathways adapt to sustain the metabolic stress of cancer cells. This non-oncogene addiction creates new vulnerabilities to target [6]. One of these stress responses is the adaptation of the lysosomal compartment. Lysosomes are acidic organelles in the cytoplasm responsible for the degradation of biological macromolecules and are also involved in lipid metabolism, membrane repair and cell death [7]. Many cancer cells have an increased number of lysosomes to support survival in stress conditions [8]. An option to target the lysosomes of cancer cells is lysomotropic drugs, which are amphiphilic drugs that have basic pKa values. With their hydrophobic molecular structure, they pass the lysosomal membrane and accumulate in the lysosome, resulting in an increased pH within the compartment, impairing lysosomal functions. Another option to target lysosomes is the inhibition of lysosomal sphingolipases, as lysosomal lipases are responsible for hydrolyzing sphingolipids in the lysosome [9]. In fact, the sphingolipid metabolism is dysregulated in many cancers, and the altered levels of pro-apoptotic and anti-proliferative lipids, such as ceramide and sphingosine-1-phosphate, contribute to tumorigenicity in cancer [10]. Fluoxetine and amitriptyline, which are approved for major depressive disorder, inhibit the acid sphingomyelinase and thus can be applied to target lysosomes.

The identification of functional vulnerabilities is currently implemented in pediatric precision oncology programs, such as ‘individual Therapies’ (iTHER), Zero Childhood Cancer (ZERO) and INdividualized Therapy FOr Relapsed Malignancies in Childhood (INFORM) through the use of patient-derived tumor samples, which are ex vivo measured for the cellular response to drugs [11–13]. Together with comprehensive genomic profiling, the drug sensitivity profiling aims to identify personalized treatment options for children with high-risk tumors [11–13]. Notably, as drug responses can range from cell death to cell cycle arrest and therapy-induced senescence, pure metabolic activity assays to detect the cellular response to drug treatment might not always be sufficient. Hence, image-based high-content drug screens with machine learning supported analysis tools are proposed to add additional layers of information [14, 15]. Therapy-induced senescence is characterized by a proliferative arrest and a senescence-associated secretory phenotype (SASP). Therapy-induced senescence plays a pivotal role in the clinical treatment of neuroblastomas, as it has been observed in response to chemotherapy in neuroblastoma cells.

Here, we aimed to identify specific vulnerabilities in neuroblastoma subtypes to address the limited treatment options for relapsed neuroblastomas, particularly those with mesenchymal features. We applied high-throughput and high-content drug screens to a diverse panel of neuroblastoma models, including patient-derived fresh tissue cultures. This revealed that MEK inhibitors, and especially the combination of MEK inhibitors with senolytics, are promising options for relapsed neuroblastomas.

## Material and methods

### Cell culture and patient-derived fresh tissue culture

The drug sensitivity profiles of the 91 cell models were obtained from multiple collaboration projects; the accession number, or if not available, the projects and labs of origin are listed in Suppl. Table 1. Cells were regularly checked for absence of bacterial and viral contamination (including several mycoplasma strains, SMRV and EBV) and authenticated by DNA fingerprinting (Multiplexion, Heidelberg, Germany). Primary cells were obtained as part of the INFORM registry and isolated from fresh tumor tissue as described by Peterziel et al. [11]. Tumor pieces were homogenized and enzymatically digested, the released DNA was digested with DNase I, and the cell suspension was passed through a strainer to obtain single cells in solution. Cells were kept in serum free tumor stem medium (Neurobasal A (1:2), DMEM-f12, (1:2) supplemented with HEPES Buffer (1:100), MEM sodium pyruvate (1:100), MEM non-essential amino acids (1:100), L-Glutamine (1:100) Penicillin–Streptomycin solution (1:100), B27 Supplement minus vitamin A (1:50), H-EGF (1:5000), H-FGF (1:5000), H-PDGF-AA (1:2000)) as tumor spheroid cultures and subcultured by dissociation with TrypLE express (Life Technologies) and seeded in fresh media. When the cell culture surpassed ex vivo passage six, the culture was considered a patient-derived long-term culture (LTC). Ex vivo fresh tissue culture screens were performed with material obtained through INFORM from the following patients: #1 (INF_R_1551, 3-year-old boy, born 2015, CDKN2A/B deletion); #2 (INF_R_2359, 7-year-old boy, born 2015); #3 (INF_R_1506, 11-year-old boy, born 2009, ALK (R1275Q) and AKT3 (E17K) mutation); #4 (INF_R_359, 9-year-old boy, born 2012, PIK3CA (H1047R) mutation, MYCN amplification,); #5 (INF_R_2077, 7-year-old boy, born 2014, MYCN amplification). For the zebrafish embryo xenograft: INF_R_1632, 3-year-old boy, born 2017, MYCN amplification, PTPN11 (E76A) mutation.

### Written informed consent statement and ethical approval for INFORM samples

The study was conducted in accordance with Good Clinical Practice guidelines and the Declaration of Helsinki. All patients, their legally acceptable representatives, or both (if possible) provided written informed consent. Approval for the study protocol (and any modifications thereof) was obtained from independent ethics committees and the institutional review board at each participating center. The study was registered with the German Clinical Trial Register, number DRKS00007623.

### Drugs and drug library

The drug library for the metabolic screens is described in Peterziel et al. [11]. The library used in the morphological screens is an extended version of this library and includes additional drugs as listed in Suppl. Table 2. Drugs were administered onto the U-shaped 384-well plates (Corning 3985, Corning, NY, USA) with Tecan D300 drug dispenser (Tecan, Männedorf, Switzerland). For repeated screening experiments, source plates were prepared with the Tecan D300 and assay plates were generated from source plates using the Mosquito LV liquid handling system (SPT Labtec, Melbourn, Hertfordshire, UK). Additional experimental drugs, such as lysosomal inhibitors and senolytics, are listed in Suppl. Table 3.

### Metabolic activity assay

The drug screening and metabolic activity assays were done as described previously [11]. Briefly, 1000 cells/well were seeded in 25 µL of respective media into the plate and centrifuged (206 rcf, 3 min) to accumulate them at the bottom of the plate for spheroid formation. After 72 h incubation, 15 µL of Cell Titer Glo 2.0 (Promega, Madison, WI, USA) was added to each well and incubated on a shaker for 20 min before luminescence was measured with a PHERAstar FSX plate reader (BMG Labtec, Ortenberg, Germany). Raw luminescence signal data were analyzed with iTreX [16]. A five-parameter dose response curve was fitted to the data points, and a drug sensitivity score (DSS_asym_) based on an asymmetric curve fit was calculated [11, 16, 17].

### Immunofluorescence staining

The cells were grown in their respective culture media (Suppl. Table 1) and fixed in the well with 4% PFA for 30 min at RT, permeabilized with 0.1% TritonX for 10 min at RT and blocked for 1 h with 3% BSA, 0.005% TritonX in PBS. Primary antibodies were diluted in 3% BSA, 0.005% TritonX in PBS added to the plate and incubated overnight at 4 °C (LAMP1 1:1000, # H3A4; LAMP2 1:1000 # sc-18822; YAP1 1:200 # 14074 T; SNAI2 1:200 #9585 T, Cell Signaling, Danvers, MA, USA; PRRX1 1:200, #nbp2-68808, Novus/R&D, Centennial, CO, USA; 2H3 anti-neurofilament (NF-M)—Developmental Studies Hybridoma Bank (DSHB), Iowa City, Iowa, USA). After washing three times with PBS, cells were incubated for 1 h at room RT with the secondary antibody of the respective species (anti-mouse IgG, Alexa488, #4408S, Donkey anti-Rabbit IgG, Alexa 568, #A10042) diluted in 3% BSA, 0.005% TritonX in PBS to a final concentration of 1:2000. After washing, the plates were imaged with the High-Content-Microscope IXM-confocal, Molecular devices, San Jose, CA, USA (HCM).

### Lysotracker staining

Cells were grown in their respective culture media (Suppl. Table 1) and kept at 37 °C for 24 h and stained with Hoechst 33342 (1:10,000), Lysotracker red DND-99 (LT) (Invitrogen, Darmstadt, Germany) (1:10,000) and Cell Mask (Invitrogen, Darmstadt, Germany) (1:4000) for 30 min at 37 °C and imaged with the HCM, with 4 sites per well with 20**×** or 40**×** magnification.

### Flow cytometric cell cycle analysis

Cells were treated with solvent or trametinib for 72 h, trypsinized for 3 min, washed in PBS and fixed with ice-cold 70% ethanol for 30 min. After washing with PBS and resuspending in 50 µL PBS, 200 µL of 50 µg/mL propidium iodide staining (#J66584.AB Thermo Fisher Scientific Inc.) was added. Fluorescence intensity was measured with a BD Canto II flow cytometer (BD Biosciences) with a band-pass 584/42 filter set. Cell cycle phase distribution was analyzed with the FlowJo software version 10 cell cycle analysis tool.

### Relative real-time reverse transcription PCR

For determination of mRNA levels, 10^6^ cells were collected, and RNA was isolated with the RNeasy Mini Kit (#74106, QIAGEN, Hilden, Germany) according to the manufacturer’s instructions, including optional on-column DNase digestion (RNase-free DNase, #79254, QIAGEN). One microgram of RNA was reverse transcribed to cDNA using the First-Strand cDNA Synthesis Kit (#K1612, Thermo Fisher Scientific Inc.) following the manufacturer’s instructions. For real-time PCR, 25 ng cDNA and 0.4 μm each of forward and reverse primers were added to Platinum™ SYBR™ Green qPCR SuperMix-UDG (#11733038 Invitrogen), and amplification was measured using a 7500 Real Time PCR System by Applied Biosystems (Thermo Fisher Scientific Inc.). Thermocycling conditions were 2 min at 50 °C, 10 min at 95 °C, 40 cycles of 15 s at 95 °C and 1 min at 60 °C, and 30 s at 95 °C and 15 s at 60 °C. The following primers were used: *CDKN1A* (p21) forward TGG AGA CTC TCA GGG TCG AAA; *CDKN1A* (p21) reverse GGC GTT TGG AGT GGT AGA AAT C; HPRT forward: 5′-TGA CAC TGG CAA AAC AAT GCA-3′; *HPRT* reverse: 5′-GGT CCT TTT CAC CAG CAA GCT-3′; *SDHA* forward: 5′-TGG GAA CAA GAG GGC TGC TG-3′; *SDHA* reverse: 5′-CCA CCA CTG CAT CAA ATT CAT G-3′. *SDHA* and *HPRT* were used as housekeeping genes, and fold changes were calculated based on the 2^−ΔΔCT^ method [18].

### Western blotting

For western blotting, 10^6^ cells were seeded in 10 cm dishes and treated for 6 h. Cells were lysed on ice for 20 min in NP-40 buffer (50 mm Tris, pH 8, 150 mm NaCl, 1% NP-40) with phosphatase inhibitors (PhosSTOP EASYPack, #04906845001, Roche Diagnostics GmbH, Mannheim, Germany) and protease inhibitors (cOmplete protease inhibitor cocktail, #11697498001, Roche Diagnostics GmbH). Debris was pelleted by centrifugation at 20,000×*g* for 15 min at 4 °C, and the supernatant was collected. Before SDS PAGE, 4× Laemmli (62.5 mm Tris, pH 6.8, 20% glycerol, 4% SDS, 5% beta-mercaptoethanol, bromophenol blue) was added to the sample, followed by incubation at 95 °C for 5 min.

The following primary antibodies were used: mouse anti-GAPDH (#MAB374, Merck KGaA), rabbit anti-ERK (#4695S, Cell Signaling, Danvers, MA, USA), and rabbit anti-pERK (T202/Y204) (#4370S, Cell Signaling). Secondary antibodies: donkey anti-rabbit IgG HRP (#31.458, ThermoFisher Scientific Inc.) and goat anti-mouse IgG HRP (#115-035-003, DIANOVA, Hamburg, Germany). The Precision Plus Protein™ Kaleidoscope™ Prestained Protein Standard (#1610375, Bio-Rad, Munich, Germany) was loaded to monitor protein separation and estimate the molecular weight of sample proteins. The complete images of all Western blots shown are included in the supplemental material. Image quantification was performed in ImageJ (version 1.53e; National Institutes of Health, Bethesda, MD, USA).

### High-content imaging assays

For basal lysosome imaging, cells were seeded in a 96-well half-area plate (Corning, NY, USA) at different cell numbers according to the cell size (2500-3500 cells/well) in 50 µL of their respective culture medium. LT staining was performed as described above and without removing the staining media. The cells were fixed in the well with 4% PFA for 30 min at RT. Each plate was imaged with the HCM, with 4 sites per well, with 40× magnification in the respective channels. After the first imaging round, immunofluorescence (IF) staining was performed as described above with LAMP1 or LAMP2 antibody at a final concentration of 1:1000. To calculate the combined lysosomal score, the scores of the three lysosomal measurements (LAMP1, LAMP2, LT) for each cell line were normalized to the Gi-M-EN cell line for each plate, to correct for plate effects, and the means for each measurement were summed up for each cell line.

For functional lysosomal adaptation, cells were seeded into flat-bottom 384-well plates (2500 cells/well) and drugs were dispensed with a Tecan D300 drug printer. After incubation for 72 h, plates were stained and imaged as described above. To compare lysosomal adaptation to drug treatments between cell lines, the number of lysosomes per image was divided by the number of nuclei per image and normalized to the DMSO-treated control. For each of the five drug concentrations, the difference from the DMSO control was summed up to obtain a lysosomal adaptation score.

### Analysis of high-content imaging assay

For lysosomal measurements, images were analyzed with a Cell Profiler (version 4.2.6) pipeline [19]. Nuclei and cells were segmented according to Hoechst and Cell Mask stains, respectively. Lysosomes were segmented based on the lysosomal stains. Cell and lysosomal dimensions and relations between them were calculated, and the intensity was measured. For classification of YAP1 immunofluorescence staining, nuclei were segmented in the images and intensity and morphological features were measured with Cell Profiler. Nuclei were classified with the machine learning based Cell Profiler Analyst classification tool [20].

### Senescence assay

The senescence β-galactosidase staining kit (Cell Signaling, Denver, MA, USA) and CellEvent Senescence Green Detection kit (Invitrogen, Darmstadt, Germany) were applied according to the manufacturer’s instructions.

### Single sample gene set enrichment analysis (ssGSEA) and mesenchymal/adrenergic (mes/adr) score

Calculation of ssGSEA scores was adapted from Barbie et al. [21]. The mesenchymal/adrenergic (mes/adr) score is calculated from the top 10 neuroblastoma super enhancers assigned to the mesenchymal identity that were identified in a genome-wide H3K27ac profiling (ChIP-seq for H3K27ac marks) by Gartlgruber et al. [5]. This super enhancer-driven signature and its corresponding transcriptional core regulatory circuitries (CRCs) direct distinct gene expression programs that define the subtype identity. Cell lines were additionally classified into mes, adr and non-defined based on chromatin-immunoprecipitation sequencing (ChIP-seq) by Gartlgruber et al. [5]. The gene expression data for the neuroblastoma cell line cohort (F. Westermann, ensh37e75) and the INFORM patient cohort (ps_inform_pedinform1056_u133p2_box1635450459) were obtained from the R2 platform. The list of SASP-associated gene signatures was obtained from Coppe et al. [22]; the MAPK pathways activity score (MPAS) gene list from Wagle et al. [23], MEKi sensitivity score (MSS) from Sigaud et al. [24], Gene Ontology (GO) senescence from MSigDB [25].

### Zebrafish

#### Zebrafish lines and maintenance

The care for and breeding of the zebrafish were performed under standardized conditions, as described previously [26]. The zebrafish wild-type AB line was raised at 28 °C. Embryos used for tumor injections were maintained in an E3 buffer (292.2 mg/L NaCl; 12.6 mg/L KCl; 36.3 mg/L CaCl2; 39.8 mg/L MgSO_4_) supplemented with 0.2 mM 1-phenyl-2- thiourea (PTU, Sigma).

#### Cell preparation and zebrafish xenotransplantation

Mesenchymal SH-EP neuroblastoma cells were cultured to 80% confluence, then washed once with PBS (Lonza, Basel, Switzerland) and harvested using trypsin (Gibco). The total number of cells and viability were determined by using an automated Vi-Cell XR Cell Viability Analyzer (Beckman Coulter, Krefeld, Germany). Cells were centrifuged (400×*g*, 5 min) and the pellet was resuspended in 5–10 mL of phenol red-free RPMI media. Cells were stained using a concentration of 5 µL × 10^6^ cells of CellTracker CM-DiD (Thermo Fisher Scientific, Waltham, MA, USA) for 5 min at 37 °C and then 10 min on ice covered from light. After centrifugation, cells were washed with serum-free RPMI and put through a 40 μm cell strainer (Greiner 542040). Cells were then centrifuged again and resuspended in serum-free RPMI to a final concentration of 5 μl × 10^6^ cells. Embryos were maintained until 2 days post fertilization at 28 °C. Only dechorionated embryos were selected, anesthetized with 1× Tricaine (0.02%, Sigma), and embedded in 1% agarose (Sigma). For the injections, 20–23 µm needles (BioMedical Instruments, Germany) were used, mounted into a micromanipulator and connected to a FemtoJet express microinjector (Eppendorf, Hamburg, Germany). Cells were injected into the yolk sac of the zebrafish embryo. After 24 h maintained at 34 °C, embryos were checked, and the ones having tumors were imaged with the HCM aligned in the Hashimoto plates (HDK-ZFA101-02a, Funakoshi, Japan). Drug treatment with navitoclax 10 μM and trametinib 250 nM (previously tested for toxicity) was performed for 48 h. At 72 h post injection (hpi) early larvae were imaged again. Tumor volume was analyzed with the FIJI software. Statistical analysis was performed with Prism 8 (Graphpad, USA). We used the non-parametric Mann–Whitney test to compare control with treated embryos.

### Graphics and statistics

Graphical representations were done with R version 4.3.2 and were plotted with R package ggplot2 3.4.4 [27] and its extension for imputation of statistical and correlation analysis ggpmisc 0.5.5, heatmaps were done with pheatmap 1.0.12 and ComplexHeatmap 2.18.0 [28]. Principal component analysis (PCA) was done with the R package factoextra 1.0.7. To calculate the quantile rank, the DSS of all cell lines in the cohort for a drug were ranked, and the sum of DSS below the cell line of interest is divided by the total number of cell lines. If a drug effect had a quantile rank above 75% it was classified as a hit; if a drug effect had a quantile range above 95%, it was classified as a top hit. BioRender was used for the generation of illustrations.

## Results

### Mesenchymal neuroblastoma cell lines are specifically sensitive to MAPK inhibition

**Fig. 1:**
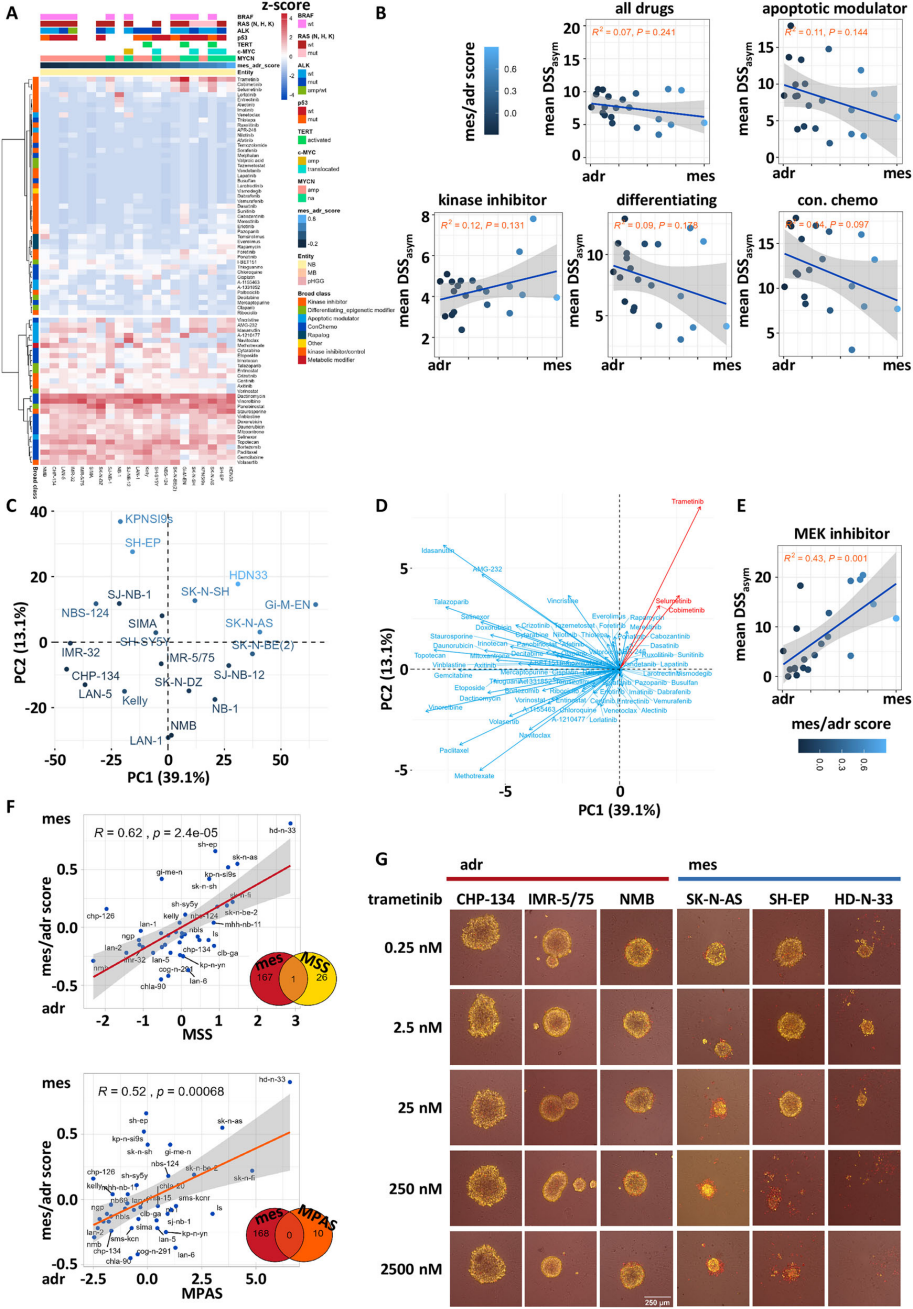
Mesenchymal neuroblastomas are specifically vulnerable to MEK inhibitors. **A** Heatmap of *z*-score of quantile-ranked drug sensitivity scores (DSS_asym_) based on metabolic activity for all 24 tested neuroblastoma cell lines to the drug library of 75 drugs. Cells were cultured as 3D spheroids and treated for 72 h. Mesenchymal-adrenergic score and molecular alterations for all neuroblastoma cell lines are indicated in the top annotation. **B** Pearson’s correlation between mes/adr score and mean DSS_asym_ of 24 neuroblastoma cell lines. Correlations were calculated for all drugs, apoptotic modulators, kinase inhibitors, differentiating agents and conventional (con.) chemotherapeutics. Dots represent cell lines and are colored according to the mesenchymal-adrenergic gene signature score. **C** Principal component analysis (PCA) performed on DSS_asym_ of 24 neuroblastoma cell lines. Dots represent cell lines and are colored according to the mesenchymal-adrenergic gene signature score (see (**B**)). **D** PCA Biplot, loading arrows represent the weight each drug response has on PC1 and PC2. **E** Pearson’s correlation between mes/adr score and mean DSS_asym_ of 24 neuroblastoma cell lines for MEK inhibitors. Dots represent cell lines and are colored according to the mesenchymal-adrenergic gene signature score (see (**B**)). Crossbars represent geometric means, and the classes were compared using the Wilcox rank sum test. **F** Scatter plot showing correlation between MAPK inhibitor sensitivity (MSS) and MAPK activity (MPAS) and mesenchymal-adrenergic score of a gene expression data set of 39 neuroblastoma cell lines. Venn diagrams display hardly any overlap between the gene signatures. **G** Example images of tumor spheroids of six neuroblastoma cell lines (*n* = 3 adrenergic and *n* = 3 mesenchymal) treated with increasing concentrations of trametinib for 72 h. Viable cells are stained with the mitochondrial membrane potential indicator TMRE (yellow) and dead cells with reddot (red), scale bar 250 µm.

We established a database with results from drug sensitivity profiling (DSP) of pediatric cell lines, including INFORM patient-derived long-term tumoroid cultures, using a library of 76 cancer-relevant drugs in clinically relevant concentrations (Fig. 1A, Suppl. Fig. 1). The database, which is continuously expanded, currently comprises DSP results of 91 cell culture models and allows cohort-based response analyses (COBRA) enabling to identification of above-average or pronounced outlier responses, in class effects and diagnosis-specific response patterns. Analyzing the COBRA DSP cohort revealed clustering mainly according to drug classes, e.g., chemotherapeutics, apoptotic modulators and kinase inhibitors (Fig. 1A, Suppl. Fig. 1B). The deeper analysis of the sensitivity of a panel of 24 three-dimensionally (3D) cultured neuroblastomas revealed a similar pattern (Fig. 1; Suppl. Figs. 2 and 3). The classification for the epigenetically regulated mesenchymal and adrenergic subtypes as published by Gartlgruber et al. [5]. was used to compare drug sensitivities of the subtypes. The classification was confirmed by immunofluorescence staining of mesenchymal markers (Suppl. Fig. 1C). As adrenergic and non-adrenergic cells are postulated to react differently to certain treatment options, we focused on the comparison of neuroblastoma adrenergic versus mesenchymal for the four main drug classes: ‘apoptotic modulator’, ‘conventional chemotherapy’, ‘kinase inhibitors’ and ‘differentiating/epigenetic modifier’. The mean drug sensitivity score (DSS_asym_) [17] over all drugs revealed no significant difference between neuroblastoma subclasses. Furthermore, most cell lines, regardless of how they were classified, were rather insensitive towards apoptosis inducers and kinase inhibitors. Only a trend toward higher sensitivity for adrenergic cell lines for differentiating compounds and for chemotherapeutics was visible (Fig. 1B). To further characterize the diverse drug response of adrenergic versus mesenchymal cells, we applied a principal component analysis (PCA) to the acquired drug sensitivity profiles. Mesenchymal cell lines grouped together, indicating a similarity of their profiles (Fig. 1C). By overlaying the loadings to visualize the influence of each individual drug response to the variance of the principal components (PCs), it revealed that all three MEK inhibitors of the library, trametinib, selumetinib and cobimetinib, had a strong weight in PC1, with trametinib being the strongest one (Fig. 1D). The close angle between the three MEK inhibitor vectors indicated a stronger correlation among these variables compared to the others. This was also reflected in a significant difference for the mean DSS_asym_ values for MEKi (Fig. 1E). These data outline a sensitivity for MEK inhibition in mesenchymal neuroblastomas and that MAPK signaling might be linked to the mesenchymal phenotype and not any other molecular feature (Fig. 1A). To address this further, we used the MAPK inhibitor sensitivity score (MSS), a gene signature indicative of MAPK pathway inhibitor sensitivity [24] and the MAPK pathway activity score (MPAS), a score calculated from the expression of MAPK pathway target genes, reflecting MAPK transcriptional activity [23]. Both the MSS signature and the MPAS signature expression positively correlated with the mesenchymal signature of Gartlgruber et al. [5], which is calculated from target genes of mesenchymal super enhancers (Fig. 1F), which is in line with the increased sensitivity of mesenchymal neuroblastoma cells to MAPK inhibitors. Notably, the MSS/MPAS gene lists hardly showed any gene overlap with the mesenchymal-adrenergic gene list, indicating independence (Fig. 1F, Venn diagrams). This was further validated by ranking the drugs for individual cell lines. The three MEK inhibitors (trametinib, selumetinib, cobimetinib) present in the library ranked as hits (above 75% quantile) or even top hits (above 95% quantile) for the more mesenchymal lines SK-N-AS, GI-M-EN, and KP-N-SI9s, but not for the more adrenergic lines IMR-32, CHP-134, LAN-5, and NB-1 (Suppl. Fig. 4). Notably, chemotherapeutic drugs, such as methotrexate and dactinomycin, classified as hits for the adrenergic lines, but not for the mesenchymal lines. The cell lines with *ALK* aberrations (SH-SY5Y, NB-1, LAN-5) indicated ALK inhibitors (e.g., lorlatinib) as hits and hence served as positive controls for the drug screening assay including the bioinformatic analysis pipeline (Suppl. Figs. 4 and 5). The cell lines SH-EP (mes) and SH-SY5Y (adr) are sublines, derived from the cell line SK-N-SH, which consists of both subtypes [29]. For the mesenchymal SH-EP cells, two of the three MEK inhibitors classified as hits, whereas in the adrenergic SH-SY5Y none of them was a hit (Suppl. Fig. 5A). The quantile score significantly separated adrenergic-type from mesenchymal-type cells (Suppl. Fig. 5B). In addition to the metabolic data, spheroid shrinkage was measured in images and confirmed the strong sensitivity of mesenchymal neuroblastoma cells for MEKi, indicative of cell death (Fig. 1G; Suppl. Fig. 5C). Furthermore, MAPK activity and on-target activity with pERK/ERK was confirmed by Western blot (Suppl. Fig. 5D and Supplementary Material).

Overall, the systematic high throughput screening of cell lines reflecting the range between adrenergic to mesenchymal neuroblastoma cell lines, revealed a specific sensitivity of mesenchymal neuroblastoma cell lines to MEK inhibitors, confirmed by higher expression of the MAPK inhibitor sensitivity and the MAPK pathway activity gene signature.

### Mesenchymal neuroblastoma cell lines and fresh tissue cultures are primed for senescence

**Fig. 2:**
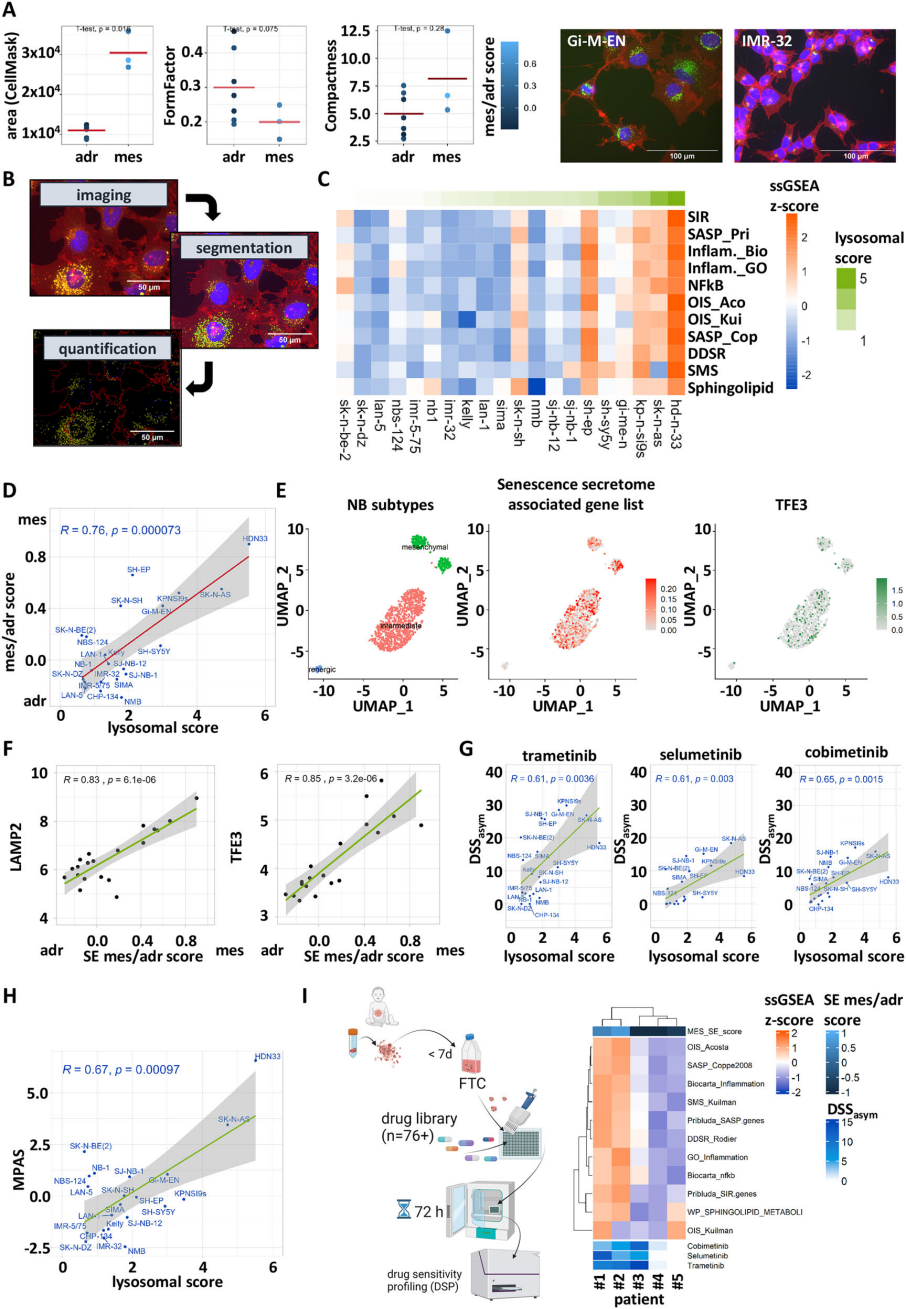
Mesenchymal neuroblastomas show characteristics of senescence. **A** Left: Comparison of phenotypic features measured with Cell Profiler-based image analysis of cells stained with Hoechst (Nuclei), CellMask (Cell membrane), Lysotracker (functional lysosomes) and LAMP1 (Lysosomal membrane protein) of mesenchymal and adrenergic cells. Feature measurements are the mean of six replicates. Right: Exemplary images of the mesenchymal (Gi-M-EN) and adrenergic (IMR-32) phenotype. Scale bar 100 µm. Nuclei are colored in blue, Cell membranes in red and lysosomes in green. **B** Segmentation based on cellular and lysosomal staining and feature extraction from segmented objects in the high-content imaging pipeline. Scale bar 50 µm **C** ssGSEA of senescence secretory phenotype-associated pathways in correlation with microscopically determined lysoscore (green color). calculated from the sum of three lysosomal measurements normalized to the Gi-M-EN cell line. Gene sets: SIR: Pribluda_SIR.gense. SASP_Pri: Pribluda_SASP.genes. Biocarta_Inflammation. GO_Inflammation. Biocarta_nfkb. OIS_Acosta. OIS_Kuilman. SASP_Coppe2008. DDSR_Rodier. SMS_Kuilman. WP_SPHINGOLIPID_METABOLISM_IN_SENESCENCE. **D** Pearson’s correlation between mes/adr score and lysosomal score of 24 neuroblastoma cell lines. **E** Uniform manifold approximation and projection (UMAP) embedding of single cell data of the cell line SK-N-SH published by Jansky et al. 2021. Neuroblastoma subtypes are annotated on the left plot based on Jansky et al. 2021. Expression of SASP-associated genes is shown in the middle plot; *TFE3* expression is shown on the right. **F** Pearson’s correlation of mes/adr score and gene log2 expression of *LAMP2* and *TFE3.* **G** Correlation of the lysosomal score and the DSS_asym_ for MEK inhibitors. **H** Correlation of the MPAS score and the lysosomal score. **I** Left: Schematic description of the workflow for processing, preculturing and drug sensitivity profiling of patient-derived fresh tissue samples obtained through the INFORM program. Right: Heatmap representing the ssGSEA scores for gene sets linked to senescence-associated secretome signaling in five INFORM fresh tissue patient samples, DSS_asym_ scores for three MEKi in the bottom annotation.

To complement the metabolic activity results with further information about phenotypic cell changes, we analyzed various morphological features, such as compactness, size and form factor, of treated (*n* = 8) and untreated (*n* = 31) cell lines with (*n* = 8) and without (basal; *n* = 31) treatment through confocal high-content imaging. Already in the basal state, we identified the mesenchymal cells as significantly bigger in size (area) (Fig. 2A). As an increase in cell size can indicate senescence and, especially therapy-induced senescence plays a role for neuroblastomas [28], X-gal staining of the lysosomal enzyme beta-galactosidase was applied to stain for senescent cells [30]. It revealed that doxorubicin treatment has the capability to induce senescence in SH-EP cells (Suppl. Fig. 6A). However, this stain mainly detects late-stage senescent cells and rather fails to detect a premature senescent phenotype. Therefore, we applied a fluorescent lysosomal staining using lysotracker and immune fluorescent staining of the lysosomal proteins LAMP1 and LAMP2 to our high-content screening, which allows to detect senescence in a more dynamic range compared to beta-galactosidase staining (Fig. 2B**;** Suppl. Fig. 6B). The lysosomal score calculated from the three lysosomal stains correlated with gene sets of the senescent secretory phenotype (Fig. 2C), confirming its suitability to detect senescence of neuroblastoma models, although other senescent markers, such as IL-6, are still relatively lowly expressed in neuroblastomas in comparison to senescent low-grade glioma cells (Suppl. Fig. 6C). This might indicate that the cells are primed for senescence, but are not yet fully senescent. In line with the morphological differences, the basal lysosomal score correlated (*R* = 0.76) with the mesenchymal phenotype (Fig. 2D; Suppl. Fig. 6B). We confirmed the expression of senescent related and lysosomal genes in mesenchymal and intermediate cell populations with a single nuclei RNAseq analysis of the mixed cell line SK-N-SH (Fig. 2E; Suppl. Fig. 7A). Additionally, we confirmed the different basal lysosome levels of adrenergic versus mesenchymal cells by using the isogenic mixed SK-N-SH model and combined immunofluorescence staining of the mesenchymal marker YAP1 and lysosomal marker LAMP1 (Suppl. Fig. 7B). Moreover, in bulk RNAseq, the lysosomal genes *LAMP2* and *TFE3* showed the highest correlation with the mesenchymal score (Fig. 2F). In line with the observation that mesenchymal cells present with higher MEKi sensitivity, the correlation of drug sensitivity DSS_asym_ to the lysosomal content confirmed an increased sensitivity to trametinib, selumetinib and cobimetinib for cells with a higher basal lysosomal score (Fig. 2G). Furthermore, the MAPK pathway activity, measured with the MPAS gene signature, correlated well (*R* = 0.67) with the microscopically measured lysosomal score of the respective cell lines (Fig. 2H). The international precision oncology program INFORM enrolls relapsed/refractory pediatric cancer patients, including neuroblastoma patients, for comprehensive molecular analysis and drug sensitivity profiling [11]. We analyzed the ex vivo drug sensitivity profiles from short-term fresh tissue tumoroid cultures derived from neuroblastoma patient samples obtained through the INFORM program for expression of senescence signatures and MEK inhibitor sensitivity. This confirmed the correlation of the senescent phenotype with trametinib, selumetinib and cobimetinib sensitivity (Fig. 2I). Furthermore, bulk RNAseq data of 223 neuroblastomas obtained through INFORM confirmed the upregulation of senescence signatures in samples with a higher mesenchymal score (Suppl. Fig. 7C). In line with the higher MAPK activity observed in mesenchymal cells, Gartlgruber et al. [5] showed that the activation of the MAPK pathway through the induction of H-RAS in the SK-N-BE(2)-C model with inducible HRAS V12, shifts single cells to a more mesenchymal subtype. Investigation of the SASP gene signature and *LAMP2* expression in the single nuclei dataset of that model revealed a higher expression of both in H-RAS induced cells compared to control cells (Suppl. Fig. 7D).

Overall, mesenchymal neuroblastoma cell lines not only show a high MAPK activity, but also present with high basal lysosomal content, phenotypic signs of senescence and expression of SASP-associated gene sets, indicating that mesenchymal cells in established cell lines, as well as in fresh tissue tumoroids might be primed for senescence.

### Drug treatment induces senescence in mesenchymal neuroblastoma cell lines

**Fig. 3:**
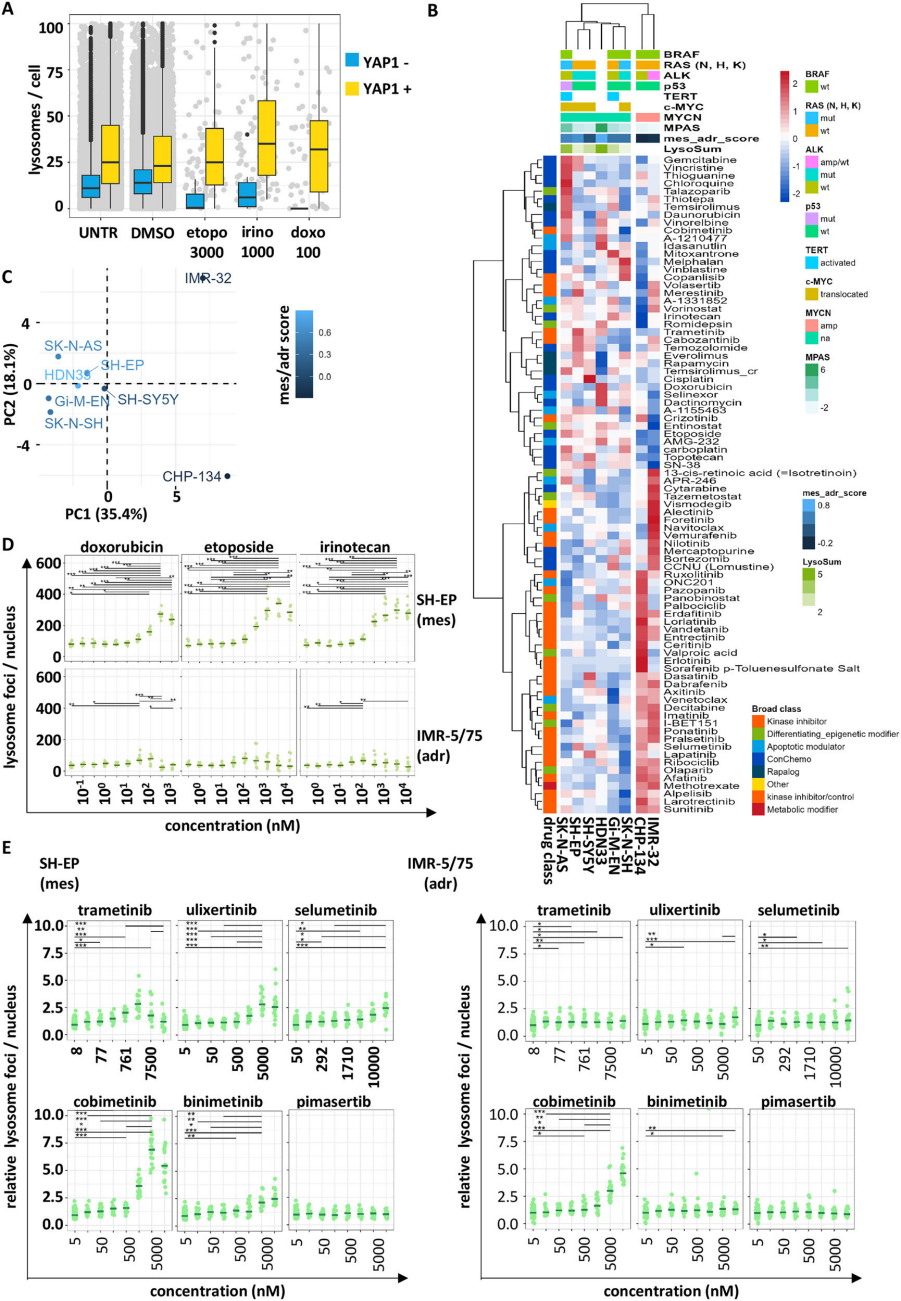
Lysosomal adaptation to drug treatment. **A** Quantification of lysosome count per cell based on immunofluorescent co-staining of YAP1 and LAMP1 in mixed cell line SK-N-SH treated with etoposide, doxorubicin and irinotecan. Number of lysosomes per cell in YAP1 staining positive and negative cells. Gray dots represent the number of individual cells. **B** Heatmap of lysosomal adaptation score of 8 neuroblastoma cell lines to 84 drugs, with genetic information of the cell lines in the top annotation. **C** PCA analysis was performed on the lysosomal adaptation score of 8 neuroblastoma cell lines. Dots represent cell lines and are colored according to the mesenchymal-adrenergic gene signature score. **D** Number of lysosomal foci per nucleus after 72 h of chemotherapy treatment with irinotecan, etoposide and doxorubicin for increasing concentrations in mesenchymal SH-EP and adrenergic IMR-5/75 cell line. **E** Number of lysosomal foci normalized to DMSO treatment for the treatment of SH-EP (left) and IMR-5/75 (right) cells with six inhibitors of the MAPK pathway. Five MEKi (trametinib, selumetinib, cobimetinib, binimetinib, pimasertib) and one ERKi (ulixertinib) were tested. Kruskal–Wallis test with post hoc Dunn comparison, non-significant comparisons not displayed, *** = padj < 0.001, ** padj < 0.01, * = padj < 0.05.

Therapy-induced senescence has been described as a neuroblastoma-relevant treatment response [28]. As our data highlights the priming of mesenchymal neuroblastoma cell lines for senescence, we analyzed the lysosomal compartment upon standard-of-care chemotherapeutic treatment for adrenergic versus mesenchymal cells in the isogenic mixed model SK-N-SH. Upon treatment with etoposide, irinotecan and doxorubicin at clinically achievable concentration (etoposide *C*_max_ 25674 nM, irinotecan *C*_max_ 1237 nM, doxorubicin *C*_max_ 116 nM [31]), lysosomes/cells substantially decreased for YAP1-negative adrenergic cells, very likely because of cell death. In contrast YAP1-positive mesenchymal cells survived and showed slight enrichment of the lysosomal compartment upon treatment compared to the DMSO control, indicative for therapy-induced senescence (Fig. 3A). After 72 h of chemotherapy treatment the YAP1 positive cells reflecting the mesenchymal subtype were enriched, which was not the case with MEKi treatment (Suppl. Fig. 13). We therefore analyzed the lysosomes per cell of a selection of 8 cell lines upon treatment for 24 h in a high-content-imaging drug screen with the extended drug library containing 84 drugs (Fig. 3B). Hierarchical clustering analysis based on the lysosomal adaptation score (fold change from DMSO) separated the cell lines by *MYCN* amplification status and mesenchymal score (Fig. 3B). This became even more evident by applying PCA to the lysosomal adaptation data, where adrenergic cells were separated from mesenchymal by dimension one (Fig. 3C). Further experiments with the mesenchymal cell line SH-EP and the adrenergic cell line IMR-5/75 demonstrated elevated lysosomal content for the mesenchymal cell line with doxorubicin, irinotecan and etoposide (Fig. 3D). The lysosomal content was up to four-fold higher compared to that of the adrenergic cell line IMR-5/75, pointing towards therapy-induced senescence in mesenchymal cells, but not in adrenergic cells (Fig. 3D). As MAPK pathway activity correlated with basal lysosomal content and mesenchymal phenotype, we investigated the lysosomal response to MAPK pathway inhibition and treated mesenchymal SH-EP and adrenergic IMR-5/75 cells with five MEK inhibitors (trametinib, cobimetinib, selumetinib, pimasertib and binimetinib) and with one ERK inhibitor (ulixertinib). MAPK pathway inhibition induced lysosomal response in the mesenchymal SH-EP cell line, but only one MEKi induced it in IMR-5/75 (Fig. 3E; Suppl. Fig. 14A). This lysosomal response to treatment increased over time and was especially evident after 72 h of treatment (Suppl. Fig. 14B). Furthermore, the lysosomal enrichment correlated with signs of senescence, identified by fluorescent beta-gal staining, change in morphology and slightly increased cell cycle arrest, indicating therapy-induced senescence (Suppl. Fig. 14C, E, F). In contrast to MEKi treatment, chemotherapy produced signs of therapy-induced senescence in both subtypes, the adrenergic and mesenchymal neuroblastoma cells (Suppl. Fig. 14F, G).

Overall, comprehensive high-content imaging screening revealed that neuroblastoma subtypes differ in their lysosomal response to MAPKi treatment. Especially, mesenchymal neuroblastomas respond with lysosome enrichment to drug treatment, potentially creating a new vulnerability. Moreover, chemotherapeutics and MAPK pathway inhibitors induce signs of senescence in mesenchymal cells, indicative for therapy-induced senescence.

### The pre-senescent phenotype creates a new vulnerability in mesenchymal neuroblastoma cells

**Fig. 4:**
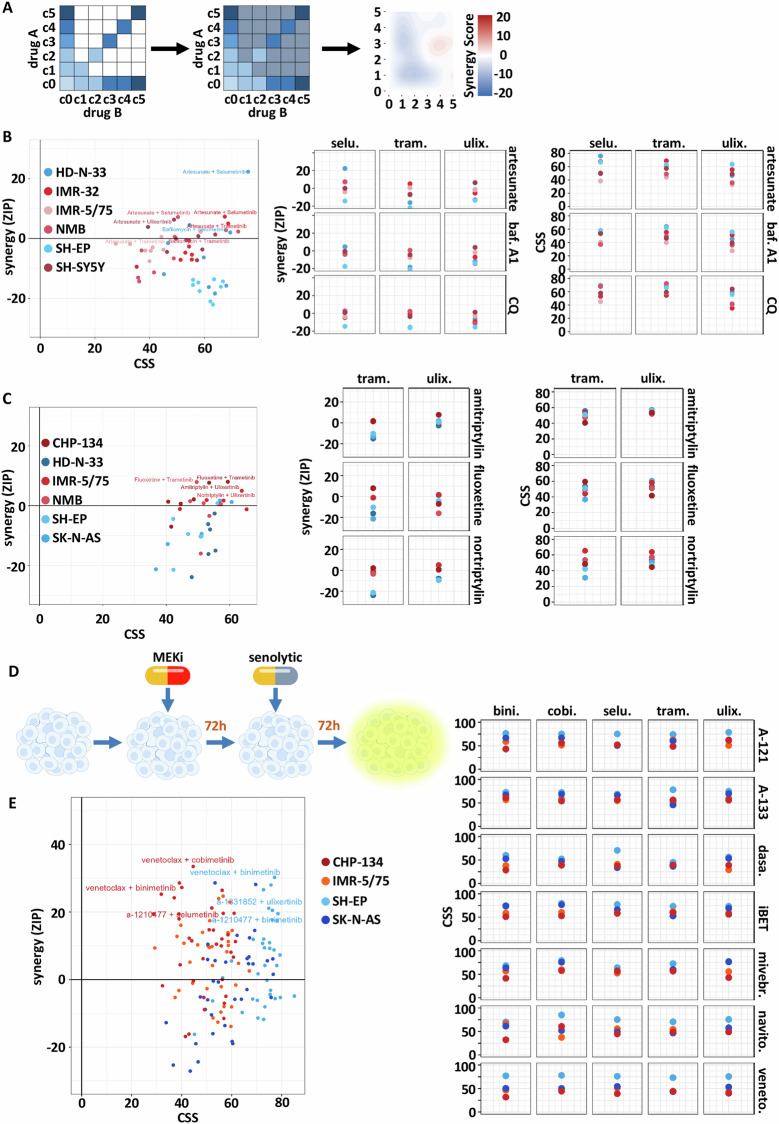
Synergy analysis of combination treatments. **A** Schematic of a diagonal minimal sampling design, blue colored squares are the concentration combinations that were measured in the combination screen. Grey colored squares were imputed, and mean synergy scores were calculated with the ZIP model. **B** Synergy-sensitivity plot of metabolic activity based, reduced matrix design synergy screen for co-treatment of lysosomal drugs (artesunate, bafilomycin A1 and chloroquine) with MAPK inhibitors (selumetinib, trametinib, ulixertinib). Two mesenchymal (HD-N.33, SH-EP) and three adrenergic cell lines (IMR-32, IMR-5/75, NMB, SH-SY5Y) were treated for 72 h. **C** Synergy-sensitivity plot of metabolic activity based, reduced matrix design synergy screen for co-treatment of sphingolipid metabolism inhibitors (amitriptyline, fluoxetine, nortriptyline) with MAPK inhibitors (trametinib, ulixertinib). **D** Graphical description of the experimental design, cells were seeded and treated with the first drug directly after seeding. After 72 h, cells were treated with senolytics. Metabolic activity readout and spheroid size analysis were done after another 72 h of incubation. **E** Synergy-sensitivity plot of metabolic activity based, reduced matrix design synergy screen for pre-treatment with senolytic drugs (A-1210477, A-1331852, dasatinib, iBET-151, mivebresib, navitoclax, venetoclax) before MAPK inhibitor (binimetinib, cobimetinib, selumetinib, trametinib, ulixertinib) treatment.

So far, our analyses revealed a specific sensitivity of mesenchymal neuroblastoma cell lines and fresh tissue tumoroids to MEK inhibitors, and a significant correlation of the mesenchymal-type with senescence phenotype-associated pathways. Hence, this lysosomal and senescent phenotype could be a novel vulnerability in the mesenchymal cells. To test this, we used lysosomal-interfering drugs, such as artesunate, bafilomycin A1 and chloroquine, as well as acid sphingomyelinase inhibitors, which are FDA approved for major depressive disorder. Furthermore, we used senolytics, such as BCL2-family inhibitors, BET inhibitors and a kinase inhibitor. With all these drugs, we performed a synergy screening with MEK inhibitor co- or pre-treated neuroblastoma cells (Fig. 4A). The combination with the classical lysosomal inhibitors was mainly additive, with effect values (CSS) mostly above 40 for adrenergic and mesenchymal cells (Fig. 4B). The FDA-approved inhibitors of lysosomal acid sphingomyelinase interfere with lysosomal membrane functions. Combinations of these inhibitors with MAPKi revealed high effect (CSS > 40), mainly attributed to additive effects, with a trend to synergy (ZIP < 10) (Fig. 4C). As treatment with MAPK inhibitors or with chemotherapy resulted in therapy-induced senescence, we pretreated the neuroblastoma cells for 72 h with either MAPK inhibitors (Fig. 4D, E) or chemotherapy (Suppl. Fig. 15), followed by another 72 h treatment with senolytics and compared it to simultaneous treatment with the drug combination for 72 h. This resulted in high synergy (ZIP up to 30) for both treatment strategies and CSS scores with mean CSS for mesenchymal cells at 70 (Suppl. Fig. 15C). CSS scores were higher for the sequential treatment strategy confirming the senescence induction in the pre-treatment (Suppl. Fig. 15). These metabolic data were also confirmed with high-content imaging of spheroid size (living area, TMRE positive) (Suppl. Fig. 16). Although mesenchymal neuroblastoma cell lines were not specifically vulnerable to single agent senolytics such as BCL-X_L_ inhibitors Navitoclax and A-1331852 (Suppl. Fig. 16B), the pre-treatment with chemotherapy rendered especially mesenchymal cells sensitive towards senolytic treatment in a synergistic manner (Fig. 4E).

**Fig. 5:**
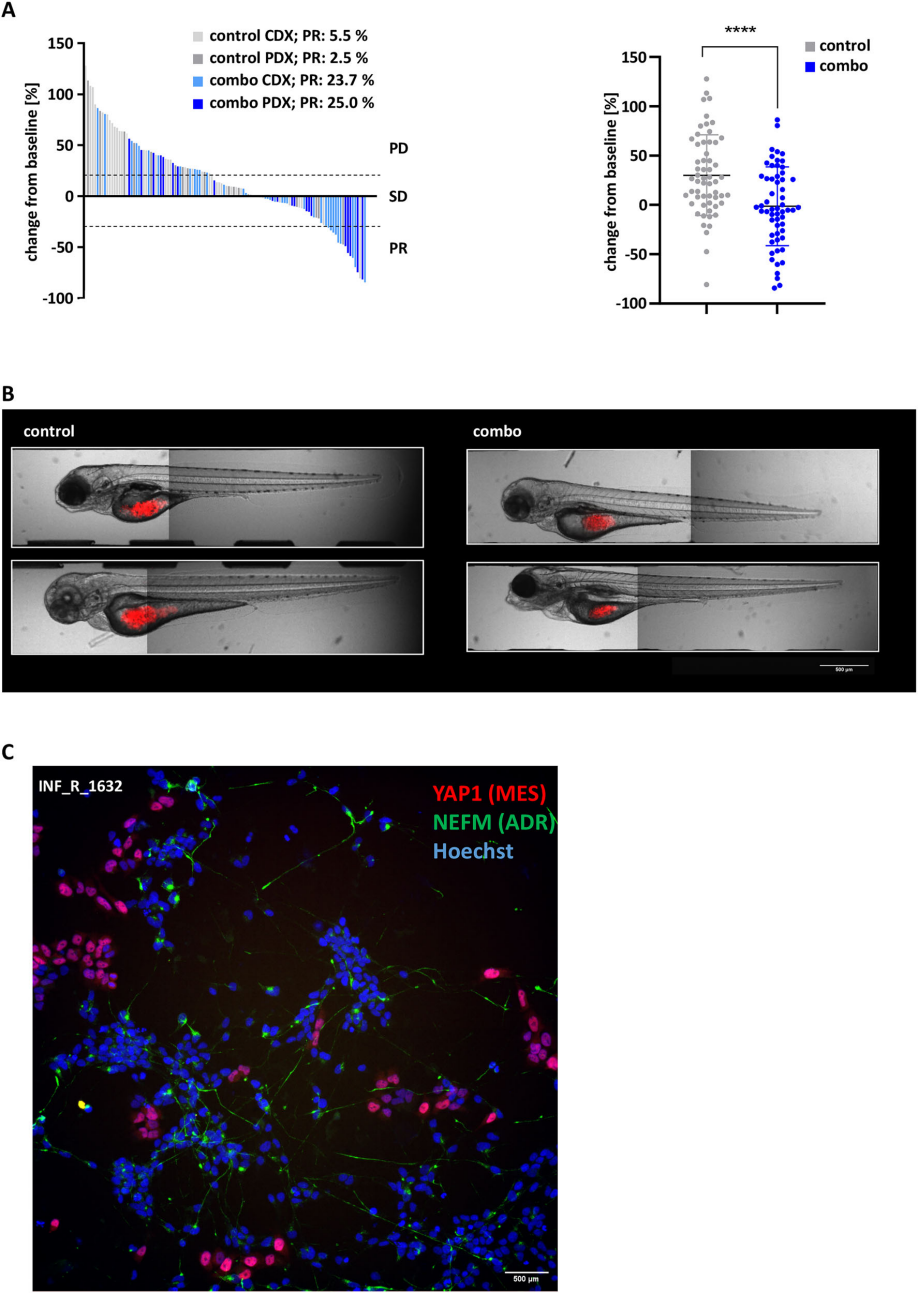
Combination treatment of navitoclax with trametinib reduces tumor growth in zebrafish xenografts. **A** Left: Waterfall plot shows the change in tumor volume (24hpi–72hpi) of each individual xenograft, treated with DMSO or trametinib (250 nM) and navitoclax (10 µM). Tumor volume was calculated based on a z-stack of confocal fluorescent images, stained with DiD. Right: Dot plot shows a significant difference in tumor volume between control and treated xenografts of four individual experiments (*n* = 78 CDX, *n* = 38 PDX). *CDX* cell line derived (SH-EP), *PDX* patient-derived (INF_R_1632_LTC). Statistical analysis was performed with the Mann–Whitney test, ****p ≤ 0.0001. **B** Representative images of zebrafish embryos xenotransplanted with SH-EP neuroblastoma cells. Brightfield images were merged with the maximum intensity projection of z-stack fluorescent images of tumor cells stained with the fluorescent cell labeling dye DiD. Early larvae were treated with navitoclax 10 µM and trametinib 250 nM for 48 h (24hpi–72hpi). Scale bar = 500 µm. **C** Immunofluorescence of patient-derived neuroblastoma cells used for zebrafish embryo injections with YAP1 (mesenchymal) and NEF-M (adrenergic) markers. The sample INF_R_1632 was obtained through INFORM. Scale bar = 500 µm.

The combination treatment of MEKi and senolytic also significantly impaired tumor growth in vivo (Fig. 5). We validated the combination of trametinib with navitoclax on neuroblastoma xenografts with both SH-EP cells (cell-derived xenograft—CDX) and patient-derived tumor cells from a 3-year-old child with a relapsed neuroblastoma enrolled in the INFORM program (patient-derived xenograft—PDX). Fluorescently labelled cells were injected into the yolk sac of the embryos and treated after 24 h for 48 h with solvent control or the treatment combination. We applied the RECIST2.0 (Response Evaluation Criteria in Solid Tumors) criteria adapted to zebrafish for the definition of partial response (PR, tumor size ≥ 30% below basal value) and progressive disease (PD, tumor size ≥ 20% above basal value) [32]. While most tumors grew progressively in the control group, we observed a significant response to the treatment in both CDX and PDX (23.7% and 25% PR) in comparison to their controls (5.5% and 2.5% PR) (Fig. 5A, B). In addition, immunofluorescence confirmed that the patient-derived tumor cells exhibited a mixed adrenergic and mesenchymal phenotype, characteristic of relapsed neuroblastomas (Fig. 5C). This is in line with the result of the fresh-tissue culture screen of a patient sample obtained from a 7-year-old boy with a relapsed neuroblastoma enrolled in the INFORM program. The patient showed resistance to the NB 2004 HR protocol in the clinic (e.g., cisplatin, etoposide, vincristine, doxorubicin, topotecan), which is also reflected in the ex vivo drug sensitivity profiling. However, the profiling of the rather mesenchymal sample indicated sensitivity for MEKi (trametinib, selumetinib, cobimetinib) and BCL2/BCL-X_L_ inhibitors (navitoclax, venetoclax) (Suppl. Fig. 17). Overall, treatment of pre-senescent mesenchymal neuroblastomas with senolytics is a promising strategy to break therapy resistance for these cells in vitro and in vivo.

## Discussion

Epigenetic subtypes add to the heterogenic clinical response of neuroblastoma, and improved treatment strategies are necessary for this complex disease. Here, we systematically analyzed drug sensitivities of a large panel (n = 91) of pediatric cell lines and patient-derived tumoroid cultures, including more than 20 neuroblastoma models, representing the range of epigenetically regulated subtypes. Neuroblastoma subtypes revealed clear differences in drug sensitivity. Specifically, mesenchymal neuroblastomas were, as expected, less sensitive to standard-of-care treatments [3, 4], but unexpectedly more sensitive towards MEK inhibitors than the adrenergic subtype. Treatment of a mixed population with standard-of-care drugs, such as etoposide, led to depletion of responsive adrenergic subtype cells and accumulation of rather resistant mesenchymal subtype cells. A shift of phenotype upon treatment could be an additional explanation for the enrichment of mesenchymal cells upon chemotherapy treatment, as the ability to transdifferentiate into one another has been observed before. Thirant et al., for example, stimulated the cells with EGF and TFNalpha to induce the transdifferentiation. This group also observed spontaneous transitions, however, over weeks of observation [30]. To unravel if short-term drug treatment could induce this transition remains to be studied. Relapsed neuroblastoma shows frequent RAS-MAPK pathway mutations, accompanied by a high degree of dysregulation in MAPK pathway activity [33–35]. Clinical trials with MEK inhibitors for neuroblastoma are ongoing (NCT06104488 and NCT02124772) in single treatment or in combination with CDK4/6 inhibitor ribociclib and BRAF inhibitor dabrafenib [36–39]. Although the MAPK pathway represents a good target for inhibition, it is very likely that cancer cells develop resistance mechanisms against the treatment, e.g., due to feedback loop regulation or interactions with other pathways [40–42]. Consequently, we investigated additional vulnerabilities of mesenchymal MEKi-sensitive neuroblastomas.

We identified a pre-senescent phenotype for the mesenchymal subtype, in which they are more susceptible to senescence-inducing stimuli. While cellular senescence is a tumor-suppressive mechanism that impairs cell proliferation in response to stress, it is also recognized that senescent tumor cells can re-enter the cell cycle to become cancer stem cells, leading to relapse after cancer chemotherapy [43]. Hence, we combined the senescence-inducing treatment with senolytic drugs that target senescence [44]. Among the senolytic drugs, BCL2-family inhibitors show the highest synergy and sensitivity scores. The BCL2 inhibitor venetoclax is currently being evaluated in Phase I trials that include both pediatric blood cancers and NB (NCT03236857). It is expected to be well tolerated in the NB setting when used in various combination regimens [45]. Notably, the adrenergic subtype showed higher synergism for the combination of MEKi and BCL2-family inhibitors, as the single drugs showed only activity in adrenergic cell lines, in contrast to mesenchymal cells. BCL2 inhibitors have shown promise in preclinical neuroblastoma models [46], particularly in combination with MEK inhibitors [47]. However, clinical development has faced challenges; navitoclax, a BCL2/BCL-X_L_ inhibitor, was discontinued due to severe side effects [48]. Venetoclax was approved for leukemia in adults and is currently evaluated for neuroblastomas in clinical trials (NCT03236857, NCT04029688), focusing on pharmacokinetics and combination therapies. The second trial was terminated due to insufficient tolerability. The BCL2 family remains a relevant target, with novel strategies such as degraders offering potential for improved therapies [49].

In a second approach, lysosomes were directly targeted with compounds increasing lysosomal pH or targeting lysosomal acid sphingomyelinase. Treatment with FDA-approved sphingomyelinase inhibitors in combination with MAPK inhibitors resulted in additive effects. Acid sphingomyelinase is a lipid hydrolase that cleaves sphingomyelin into ceramide and is located in the lysosome. It was expected that inhibition of acid sphingomyelinase would be more effective in mesenchymal neuroblastomas, which present with a higher basal lysosome count [10, 50]. However, our studies did not reveal a higher efficacy for one of the subtypes. Inhibition of acid sphingomyelinase has dual effects on tumor growth. On one hand, it prevents ceramide accumulation and hence protects cells from apoptotic cell death. On the other hand, the accumulation of sphingomyelin in the lysosomal membrane increases membrane rigidity, causing membrane destabilization [51, 52] and release of cathepsins into the cytosol, ultimately killing the cells. The detergent-like capacity of the lysosomotropic drugs that inhibit acid sphingomyelinase further supports this effect [52]. Although sphingomyelinase inhibitors were beneficial combination partners for MEK inhibitors in our approach, the successive treatment with senolytics outperformed this approach due to its more synergistic effect. Overall, comprehensive high-throughput and high-content drug screening identified novel vulnerabilities, especially for mesenchymal-like neuroblastomas, druggable by repurposing of clinically approved drugs.

## Supplementary information

Supplemental Material

## Data Availability

The datasets generated and/or analyzed during the current study are available from the corresponding author on reasonable request.

## References

[1] Körber V, Stainczyk SA, Kurilov R, Henrich KO, Hero B, Brors B, et al. Neuroblastoma arises in early fetal development and its evolutionary duration predicts outcome. Nat Genet. 2023;55:619–30. 36973454 10.1038/s41588-023-01332-y PMC10101850

[2] Qiu B, Matthay KK. Advancing therapy for neuroblastoma. Nat Rev Clin Oncol. 2022;19:515–33. 35614230 10.1038/s41571-022-00643-z

[3] van Groningen T, Koster J, Valentijn LJ, Zwijnenburg DA, Akogul N, Hasselt NE, et al. Neuroblastoma is composed of two super-enhancer-associated differentiation states. Nat Genet. 2017;49:1261–6. 28650485 10.1038/ng.3899

[4] Boeva V, Louis-Brennetot C, Peltier A, Durand S, Pierre-Eugène C, Raynal V, et al. Heterogeneity of neuroblastoma cell identity defined by transcriptional circuitries. Nat Genet. 2017;49:1408–13. 28740262 10.1038/ng.3921

[5] Gartlgruber M, Sharma AK, Quintero A, Dreidax D, Jansky S, Park YG, et al. Super enhancers define regulatory subtypes and cell identity in neuroblastoma. Nat Cancer. 2021;2:114–28. 35121888 10.1038/s43018-020-00145-w

[6] Semenova EA, Nagel R, Berns A. Origins, genetic landscape, and emerging therapies of small cell lung cancer. Genes Dev. 2015;29:1447–62. 26220992 10.1101/gad.263145.115 PMC4526731

[7] Appelqvist H, Wäster P, Kågedal K, Öllinger K. The lysosome: from waste bag to potential therapeutic target. J Mol Cell Biol. 2013;5:214–26. 23918283 10.1093/jmcb/mjt022

[8] Dielschneider RF, Henson ES, Gibson SB. Lysosomes as Oxidative Targets for Cancer Therapy. Oxid Med Cell Longev. 2017;2017:3749157. 28757908 10.1155/2017/3749157 PMC5516749

[9] Kornhuber J, Muehlbacher M, Trapp S, Pechmann S, Friedl A, Reichel M, et al. Identification of novel functional inhibitors of acid sphingomyelinase. PLoS ONE. 2011;6:e23852. 21909365 10.1371/journal.pone.0023852 PMC3166082

[10] Savić R, Schuchman EH. Use of acid sphingomyelinase for cancer therapy. Adv Cancer Res. 2013;117:91–115. 23290778 10.1016/B978-0-12-394274-6.00004-2

[11] Peterziel H, Jamaladdin N, ElHarouni D, Gerloff XF, Herter S, Fiesel P, et al. Drug sensitivity profiling of 3D tumor tissue cultures in the pediatric precision oncology program INFORM. NPJ Precis Oncol. 2022;6:94. 36575299 10.1038/s41698-022-00335-y PMC9794727

[12] Wong M, Mayoh C, Lau LMS, Khuong-Quang DA, Pinese M, Kumar A, et al. Whole genome, transcriptome and methylome profiling enhances actionable target discovery in high-risk pediatric cancer. Nat Med. 2020;26:1742–53. 33020650 10.1038/s41591-020-1072-4

[13] Langenberg KPS, Meister MT, Bakhuizen JJ, Boer JM, van Eijkelenburg NKA, Hulleman E, et al. Implementation of paediatric precision oncology into clinical practice: the Individualized Therapies for Children with cancer program ‘iTHER. Eur J Cancer. 2022;175:311–25. 36182817 10.1016/j.ejca.2022.09.001 PMC9586161

[14] Berker Y, ElHarouni D, Peterziel H, Fiesel P, Witt O, Oehme I, et al. Patient-By-patient Deep Transfer Learning for Drug-response Profiling Using Confocal Fluorescence Microscopy of Pediatric Patient-derived Tumor-cell Spheroids. IEEE Trans Med Imaging. 2022;41:3981–99. 36099221 10.1109/TMI.2022.3205554

[15] Choo N, Ramm S, Luu J, Winter JM, Selth LA, Dwyer AR, et al. High-Throughput imaging assay for drug screening of 3D prostate cancer organoids. SLAS Discov. 2021;26:1107–24. 34111999 10.1177/24725552211020668 PMC8458687

[16] ElHarouni D, Berker Y, Peterziel H, Gopisetty A, Turunen L, Kreth S, et al. iTReX: Interactive exploration of mono- and combination therapy dose response profiling data. Pharmacol Res. 2022;175:105996. 34848323 10.1016/j.phrs.2021.105996

[17] Yadav B, Pemovska T, Szwajda A, Kulesskiy E, Kontro M, Karjalainen R, et al. Quantitative scoring of differential drug sensitivity for individually optimized anticancer therapies. Sci Rep. 2014;4:5193. 24898935 10.1038/srep05193 PMC4046135

[18] Fischer M, Skowron M, Berthold F. Reliable transcript quantification by real-time reverse transcriptase-polymerase chain reaction in primary neuroblastoma using normalization to averaged expression levels of the control genes HPRT1 and SDHA. J Mol Diagn. 2005;7:89–96. 15681479 10.1016/S1525-1578(10)60013-X PMC1867502

[19] Stirling DR, Swain-Bowden MJ, Lucas AM, Carpenter AE, Cimini BA, Goodman A. CellProfiler 4: improvements in speed, utility and usability. BMC Bioinforma. 2021;22:433. 10.1186/s12859-021-04344-9 PMC8431850 34507520

[20] Dao D, Fraser AN, Hung J, Ljosa V, Singh S, Carpenter AE. CellProfiler Analyst: interactive data exploration, analysis and classification of large biological image sets. Bioinformatics. 2016;32:3210–2. 27354701 10.1093/bioinformatics/btw390 PMC5048071

[21] Barbie DA, Tamayo P, Boehm JS, Kim SY, Moody SE, Dunn IF, et al. Systematic RNA interference reveals that oncogenic KRAS-driven cancers require TBK1. Nature. 2009;462:108–12. 19847166 10.1038/nature08460 PMC2783335

[22] Coppé JP, Desprez PY, Krtolica A, Campisi J. The senescence-associated secretory phenotype: the dark side of tumor suppression. Annu Rev Pathol. 2010;5:99–118. 20078217 10.1146/annurev-pathol-121808-102144 PMC4166495

[23] Wagle MC, Kirouac D, Klijn C, Liu B, Mahajan S, Junttila M, et al. A transcriptional MAPK Pathway Activity Score (MPAS) is a clinically relevant biomarker in multiple cancer types. NPJ Precis Oncol. 2018;2:7. 29872725 10.1038/s41698-018-0051-4 PMC5871852

[24] Sigaud R, Albert TK, Hess C, Hielscher T, Winkler N, Kocher D, et al. MAPK inhibitor sensitivity scores predict sensitivity driven by the immune infiltration in pediatric low-grade gliomas. Nat Commun. 2023;14:4533. 37500667 10.1038/s41467-023-40235-8 PMC10374577

[25] Subramanian A, Tamayo P, Mootha VK, Mukherjee S, Ebert BL, Gillette MA, et al. Gene set enrichment analysis: a knowledge-based approach for interpreting genome-wide expression profiles. Proc Natl Acad Sci USA. 2005;102:15545–50. 16199517 10.1073/pnas.0506580102 PMC1239896

[26] Gatzweiler C, Ridinger J, Herter S, Gerloff XF, ElHarouni D, Berker Y, et al. Functional therapeutic target validation using pediatric zebrafish xenograft models. Cancers (Basel). 2022;14:849. 35159116 10.3390/cancers14030849 PMC8834194

[27] DuBois SG, Kalika Y, Lukens JN, Brodeur GM, Seeger RC, Atkinson JB, et al. Metastatic sites in stage IV and IVS neuroblastoma correlate with age, tumor biology, and survival. J Pediatr Hematol Oncol. 1999;21:181–9. 10363850 10.1097/00043426-199905000-00005

[28] Zanotti S, Decaesteker B, Vanhauwaert S, De Wilde B, De Vos WH, Speleman F. Cellular senescence in neuroblastoma. Br J Cancer. 2022;126:1529–38. 35197583 10.1038/s41416-022-01755-0 PMC9130206

[29] Ross RA, Spengler BA, Biedler JL. Coordinate morphological and biochemical interconversion of human neuroblastoma cells. J Natl Cancer Inst. 1983;71:741–7. 6137586

[30] Thirant C, Peltier A, Durand S, Kramdi A, Louis-Brennetot C, Pierre-Eugene C, et al. Reversible transitions between noradrenergic and mesenchymal tumor identities define cell plasticity in neuroblastoma. Nat Commun. 2023;14:2575. 37142597 10.1038/s41467-023-38239-5 PMC10160107

[31] Jamaladdin N, Sigaud R, Kocher D, Kolodziejczak AS, Nonnenbroich LF, Ecker J, et al. Key pharmacokinetic parameters of 74 pediatric anticancer drugs providing assistance in preclinical studies. Clin Pharmacol Therap. 2023;114:904–13. 37441736 10.1002/cpt.3002

[32] Wrobel JK, Najafi S, Ayhan S, Gatzweiler C, Krunic D, Ridinger J. et al. Rapid In Vivo validation of HDAC inhibitor-based treatments in neuroblastoma zebrafish xenografts. Pharmaceuticals (Basel). 2020;13:345. 33121173 10.3390/ph13110345 PMC7692187

[33] Ambros PF, Ambros IM, Brodeur GM, Haber M, Khan J, Nakagawara A, et al. International consensus for neuroblastoma molecular diagnostics: report from the International Neuroblastoma Risk Group (INRG) Biology Committee. Br J Cancer. 2009;100:1471–82. 19401703 10.1038/sj.bjc.6605014 PMC2694415

[34] Shimada H, Chatten J, Newton WA Jr, Sachs N, Hamoudi AB, Chiba T, et al. Histopathologic prognostic factors in neuroblastic tumors: definition of subtypes of ganglioneuroblastoma and an age-linked classification of neuroblastomas. J Natl Cancer Inst. 1984;73:405–16. 6589432 10.1093/jnci/73.2.405

[35] Brodeur GM, Pritchard J, Berthold F, Carlsen NL, Castel V, Castelberry RP, et al. Revisions of the international criteria for neuroblastoma diagnosis, staging, and response to treatment. J Clin Oncol. 1993;11:1466–77. 8336186 10.1200/JCO.1993.11.8.1466

[36] Johnsen JI, Dyberg C, Fransson S, Wickström M. Molecular mechanisms and therapeutic targets in neuroblastoma. Pharmacol Res. 2018;131:164–76. 29466695 10.1016/j.phrs.2018.02.023

[37] Mlakar V, Morel E, Mlakar SJ, Ansari M, Gumy-Pause F. A review of the biological and clinical implications of RAS-MAPK pathway alterations in neuroblastoma. J Exp Clin Cancer Res. 2021;40:189. 34103089 10.1186/s13046-021-01967-x PMC8188681

[38] Hart LS, Rader J, Raman P, Batra V, Russell MR, Tsang M, et al. Preclinical therapeutic synergy of MEK1/2 and CDK4/6 inhibition in neuroblastoma. Clin Cancer Res. 2017;23:1785–96. 27729458 10.1158/1078-0432.CCR-16-1131

[39] Moore NF, Azarova AM, Bhatnagar N, Ross KN, Drake LE, Frumm S, et al. Molecular rationale for the use of PI3K/AKT/mTOR pathway inhibitors in combination with crizotinib in ALK-mutated neuroblastoma. Oncotarget. 2014;5:8737–49. 25228590 10.18632/oncotarget.2372 PMC4226718

[40] Irwin MS, Naranjo A, Zhang FF, Cohn SL, London WB, Gastier-Foster JM, et al. Revised neuroblastoma risk classification system: a report from the Children’s Oncology Group. J Clin Oncol. 2021;39:3229–41. 34319759 10.1200/JCO.21.00278 PMC8500606

[41] Mulcahy Levy JM, Thorburn A. Autophagy in cancer: moving from understanding mechanism to improving therapy responses in patients. Cell Death Differ. 2020;27:843–57. 31836831 10.1038/s41418-019-0474-7 PMC7206017

[42] Galluzzi L, Pietrocola F, Bravo-San Pedro JM, Amaravadi RK, Baehrecke EH, Cecconi F, et al. Autophagy in malignant transformation and cancer progression. Embo J. 2015;34:856–80. 25712477 10.15252/embj.201490784 PMC4388596

[43] Perrigue PM, Rakoczy M, Pawlicka KP, Belter A, Giel-Pietraszuk M, Naskręt-Barciszewska M. et al. Cancer stem cell-inducing media activates senescence reprogramming in fibroblasts. Cancers (Basel). 2020;12:1745. 32629974 10.3390/cancers12071745 PMC7409320

[44] Trochet D, Bourdeaut F, Janoueix-Lerosey I, Deville A, de Pontual L, Schleiermacher G, et al. Germline mutations of the paired-like homeobox 2B (PHOX2B) gene in neuroblastoma. Am J Hum Genet. 2004;74:761–4. 15024693 10.1086/383253 PMC1181953

[45] Dalton KM, Krytska K, Lochmann TL, Sano R, Casey C, D’Aulerio A, et al. Venetoclax-based rational combinations are effective in models of MYCN-amplified neuroblastoma. Mol Cancer Ther. 2021;20:1400–11. 34088831 10.1158/1535-7163.MCT-20-0710 PMC10350345

[46] Bierbrauer A, Jacob M, Vogler M, Fulda S. A direct comparison of selective BH3-mimetics reveals BCL-X-L, BCL-2 and MCL-1 as promising therapeutic targets in neuroblastoma. British J cancer. 2020;122:1544–51. 10.1038/s41416-020-0795-9 PMC7217842 32203216

[47] Eleveld TF, Vernooij L, Schild L, Koopmans B, Alles LK, Ebus ME, et al. MEK inhibition causes BIM stabilization and increased sensitivity to BCL-2 family member inhibitors in RAS-MAPK-mutated neuroblastoma. Front Oncol. 2023;13:1130034. 36895472 10.3389/fonc.2023.1130034 PMC9990464

[48] de Vos S, Leonard JP, Friedberg JW, Zain J, Dunleavy K, Humerickhouse R, et al. Safety and efficacy of navitoclax, a BCL-2 and BCL-X(L) inhibitor, in patients with relapsed or refractory lymphoid malignancies: results from a phase 2a study. Leuk Lymphoma. 2021;62:810–8. 33236943 10.1080/10428194.2020.1845332 PMC9257998

[49] Vogler M, Braun Y, Smith VM, Westhoff MA, Pereira RS, Pieper NM, et al. The BCL2 family: from apoptosis mechanisms to new advances in targeted therapy. Signal Transduct Target Ther. 2025;10:91. 40113751 10.1038/s41392-025-02176-0 PMC11926181

[50] Bartolucci D, Montemurro L, Raieli S, Lampis S, Pession A, Hrelia P. et al. MYCN impact on high-risk neuroblastoma: from diagnosis and prognosis to targeted treatment. Cancers (Basel). 2022;14:4421. 36139583 10.3390/cancers14184421 PMC9496712

[51] Kirkegaard T, Jäättelä M. Lysosomal involvement in cell death and cancer. Biochim Biophys Acta. 2009;1793:746–54. 18948147 10.1016/j.bbamcr.2008.09.008

[52] Petersen NH, Olsen OD, Groth-Pedersen L, Ellegaard AM, Bilgin M, Redmer S, et al. Transformation-associated changes in sphingolipid metabolism sensitize cells to lysosomal cell death induced by inhibitors of acid sphingomyelinase. Cancer Cell. 2013;24:379–93. 24029234 10.1016/j.ccr.2013.08.003

