# Supplementary material for: High content-imaging drug synergy screening identifies specific senescence-related vulnerabilities of mesenchymal neuroblastomas
Source: Cell Death Dis. 2025 Aug 25;16(1):644. doi: 10.1038/s41419-025-07933-1 (PMC12379013; doi:10.1038/s41419-025-07933-1)
Supplement: Supplementary file 1 — Supplemental Material [file 41419_2025_7933_MOESM1_ESM.pdf]

Supplementary Material

Figure Legends

Suppl. Figure 1: Systematic drug sensitivity profiling of pediatric cancer cell lines. (A) Schematic workflow of metabolic activity-based drug sensitivity profiling of 109 pediatric cancer cell lines, with a pie chart showing the distribution of tumor entities across the cell lines. (**B**) Heatmap of z-score of quantile-ranked Drug Sensitivity Scores (DSS_{asym}) based on metabolic activity for all tested cell lines to the drug library of 75 drugs. Cells were cultured in 3D and treated for 72h. Tumor entity and mesenchymal-adrenergic score for all neuroblastoma cell lines is indicated in the top annotation. (**C)** Quantification of immunofluorescence intensity. NB cells were stained with antibodies against mesenchymal markers YAP1, PRXX1 and SNAI2. Epigenetic super enhancer based mesenchymal-adrenergic score is indicated in the blue color bar below the graphs. Light blue: mesenchymal-type, dark blue: adrenergic.

Suppl. Figure 2: Cohort plots of Drug Sensitivity Scores. Dot plots for each drug showing the Drug Sensitivity Scores (DSS_{asym}) for all tested cell lines and all drugs, colored by tumor entity.

Suppl. Figure 3: Scatter plots depicting the correlation of DSS_{asym} and mesenchymal-adrenergic score for each drug of the library. Each dot represents a cell line of the cohort.

Suppl. Figure 4: Waterfall plots of DSS_{asym} quantile ranks of the drug responses in comparison to the cohort for specific cell lines. 75% and 95% quantiles indicate a rank classifying a drug as hit or top hit, respectively. **(A)** Mesenchymal neuroblastoma cell lines. **(B)** Adrenergic neuroblastoma cell lines. Tr: trametinib; Se: selumetinib; Co: cobimetinib; Me: methotrexate; ActD: dactinomycin; Ale: alectinib; Cr: crizotinib; Ce: ceritinib; En: entrectinib. Lo: lorlatinib.

Suppl. Figure 5: (A) Waterfall plots of quantile ranks of the drug responses of SK-N-SH cells and the cell lines derived from its mesenchymal (SH-EP) and adrenergic (SH-SY5Y) subclones. Drug ranks marked with arrows. Tr: trametinib; Se: selumetinib; Co: cobimetinib; Me: methotrexate; ActD: dactinomycin. **(B)** Mean quantile score for three MEKi (trametinib, selumetinib, cobimetinib) of neuroblastoma cell lines classified as adrenergic or mesenchymal (n = 7 adrenergic cell lines; n = 4 mesenchymal cell lines) dashed lines indicate the 75% and 95% quantiles defining hits. Statistical analysis was performed using unpaired two tailed t-test. **(C)** Quantification of metabolic activity and spheroid area of six neuroblastoma cell lines (n= 3 adrenergic and n= 3 mesenchymal) treated with increasing concentrations of trametinib are shown as percent inhibition calculated based on DMSO (negative) and benzethonium chloride (positive) treated controls. (**D)** Western blotting against pERK and ERK with CHP-134 (adr) and HD-N-33 (mes) cells treated with increasing concentrations of MEKi trametinib. Depicted are representative blots of three biological replicates. GAPDH served as a loading control. The quantification of three experiments is shown below the blot.

Suppl. Figure 6: (A) Example images of X-gal staining of the neuroblastoma cell line SH-EP treated for 6 days with doxorubicin, the non-senescent pediatric pleomorphic xanthoastrocytoma-like cell line BT40 (negative control) and the senescent pediatric pilocytic astrocytoma cellline DKFZ-B317 (positive control), scale bar 150 µm **(B)** Number of lysosomes per cell, normalized to Gi-M-EN cells, detected with lysotracker and anti-LAMP1, anti-LAMP2 staining. **(C)** Comparison of log2 expression of SASP marker IL-6 across a pLGG dataset (ps_mkheidel_expplgg6_u133p2) and a neuroblastoma data set with mesenchymal and adrenergic cell line pairs from isogenic origin (NB_M; ps_avgpres_gse90803geo8_u133p2) and a neuroblastoma cell line (NB_C; ps_avgpres_gse28019geo24_u133p2) dataset. **(D)** Correlation of log2 expression of lysosome associated genes and mes/adr score. **(E)** Venn diagrams indicating the overlap between the gene sets

Suppl. Figure 7: (A) Uniform manifold approximation and projection (UMAP) embedding of single cell data of the cell line SK-N-SH. Neuroblastoma subtypes are annotated on the left plot based on Jansky et al 2021. Expression of SASP associated gene list, LAMP2 and TFEB is shown. **(B)** Example images of immunofluorescence co-staining with LAMP1 (yellow) and YAP1 (green) antibodies. Nuclei are stained with Hoechst (blue). Scale bar 100 µm. **(C)** Heatmap representing ssGSEA scores for SASP gene sets in 223 neuroblastoma patient samples of the INFORM cohort, samples are ordered based mes/adr score. **(D)** UMAP projection of single cell data of H-

RAS inducible and control SK-N-BE(2)-C cells separated into two clusters (left). Expression of SASP associated gene list (middle), expression of *LAMP2* gene expression (right).

Suppl. Figure 8: Representative images of LAMP1 and LAMP2 immunofluorescence staining with corresponding lysotracker staining. Immunofluorescence staining of lysosomal markers LAMP1 (green) and LAMP2 (green) and Lysotracker (yellow) to highlight lysosomes. Cell membranes were labeled with CellMask (red) and nuclei were stained with Hoechst (blue). GI-M-EN cells served as a reference cell line. Scale bar: 100 µm.

Suppl. Figure 9: Representative images of LAMP1 and LAMP2 immunofluorescence staining with corresponding lysotracker staining. Immunofluorescence staining of lysosomal markers LAMP1 (green) and LAMP2 (green) and Lysotracker (yellow) to highlight lysosomes. Cell membranes were labeled with CellMask (red) and nuclei were stained with Hoechst (blue). Scale bar: 100 µm.

Suppl. Figure 10: Representative images of LAMP1 and LAMP2 immunofluorescence staining with corresponding Lysotracker staining. Immunofluorescence staining of lysosomal markers LAMP1 (green) and LAMP2 (green) and Lysotracker (yellow) to highlight lysosomes. Cell membranes were labeled with CellMask (red) and nuclei were stained with Hoechst (blue). GI-M-EN cells served as a reference cell line. Scale bar: 100 µm.

Suppl. Figure 11: Representative images of LAMP1 and LAMP2 immunofluorescence staining with corresponding Lysotracker staining. Immunofluorescence staining of lysosomal markers LAMP1 (green) and LAMP2 (green) and Lysotracker (yellow) to highlight lysosomes. Cell membranes were labeled with CellMask (red) and nuclei were stained with Hoechst (blue). GI-M-EN cells served as a reference cell line. Scale bar: 100 µm.

Suppl. Figure 12: Representative images of LAMP1 and LAMP2 immunofluorescence staining with corresponding Lysotracker staining. Immunofluorescence staining of lysosomal markers LAMP1 (green) and LAMP2 (green) and Lysotracker (yellow) to highlight lysosomes. Cell membranes were labeled with CellMask (red) and nuclei were stained with Hoechst (blue). GI-M-EN cells served as a reference cell line. Scale bar: 100 µm.

Suppl. Figure 13: Quantification of immunofluorescence intensity. Mixed phenotype SK-N-SH cells were stained with an antibody against mesenchymal marker YAP1 upon treatment with increasing concentrations of chemotherapeutic drugs (left side) and MEK inhibitors (right side). Displayed is the number of YAP1-positive cells per condition. Untr: untreated.

Suppl. Figure 14: (A) Example images of mesenchymal SH-EP cell line treated with ERKi ulixertinib and MEKi trametinib for 72h. Nuclei = blue, Lysotracker = yellow. Scale bar 20 µm **(B)** SH-EP cell line treated with MEKi trametinib and ERKi ulixertinib for indicated time points. Number of lysosomes per nucleus was normalized to DMSO treatment for each time point. **(C)** Mean Area of the cell body segmented and quantified with CellProfiler based on the CellMask stain, 6 technical replicates. SH-EP and SH-SY5Y cells were treated with indicated trametinib concentrations for 72h. **(D)** Nuclei number following 72h trametinib treatment. CHP-134 and HD-N-33 cells were cultured in 2D and nuclei were stained with Hoechst. Data of n = 3 biological replicates. **(E)** Flow cytometry analysis of cell cycle phases with fixed and PI-stained CHP-134 and HD-N-33 cells. Cells were treated with trametinib as indicated. **(F)** Intensity of fluorescent β-gal staining for multiple concentrations of three MAPKi and one chemotherapy drug normalized to DMSO treated control. Cells were exposed to drug treatment for 6 days. **(G)** Real-time PCR analysis of p21/*CDKN1A* mRNA expression in SH-EP and SH-SY5Y cells upon treatment with doxorubicin.

Supplemental Figure 15: (A) ZIP synergy and CSS scores for each chemotherapy and senolytic drug combination, for metabolic activity readout, sequential treatment. **(B)** ZIP synergy and CSS scores for each chemotherapy or MAPKi respectively, and senolytic drug combination, for metabolic activity readout, simultaneous treatment. **(C)** Synergy-Sensitivity plot for chemotherapy and senolytic drug combinations**,** sequential treatment. **(D)** Synergy-Sensitivity plot for MAPKi or chemotherapy and senolytic drug combinations**,** simultaneous treatment. **(E)** Comparison of mean ZIP and CSS scores between neuroblastoma subtypes. Combination of chemotherapy and senolytics on the left, combination of MAPKi and senolytics on the right. Sequential or simultaneous treatment as indicated.

Supplemental Figure 16: (A) ZIP synergy and CSS scores for each tested drug combination, for spheroid area as determined by imaging. **(B)** ZIP synergy and CSS scores for each tested drug combination separated for combinations with MAPKi and chemotherapeutic drugs. **(C)** Comparison of mean ZIP and CSS scores between neuroblastoma cell lines separated by drug class.

Supplemental Figure 17: Heatmap representing the drug sensitivity (DSS quantile rank) in five INFORM fresh tissue patient samples. Red arrows indicate resistance of sample #2 to indicated drugs. Green boxes mark the MEKi trametinib, cobimetinib, selumentinib and BCL2/X_Li navitoclax and venetoclax.

Suppl. Figure 1

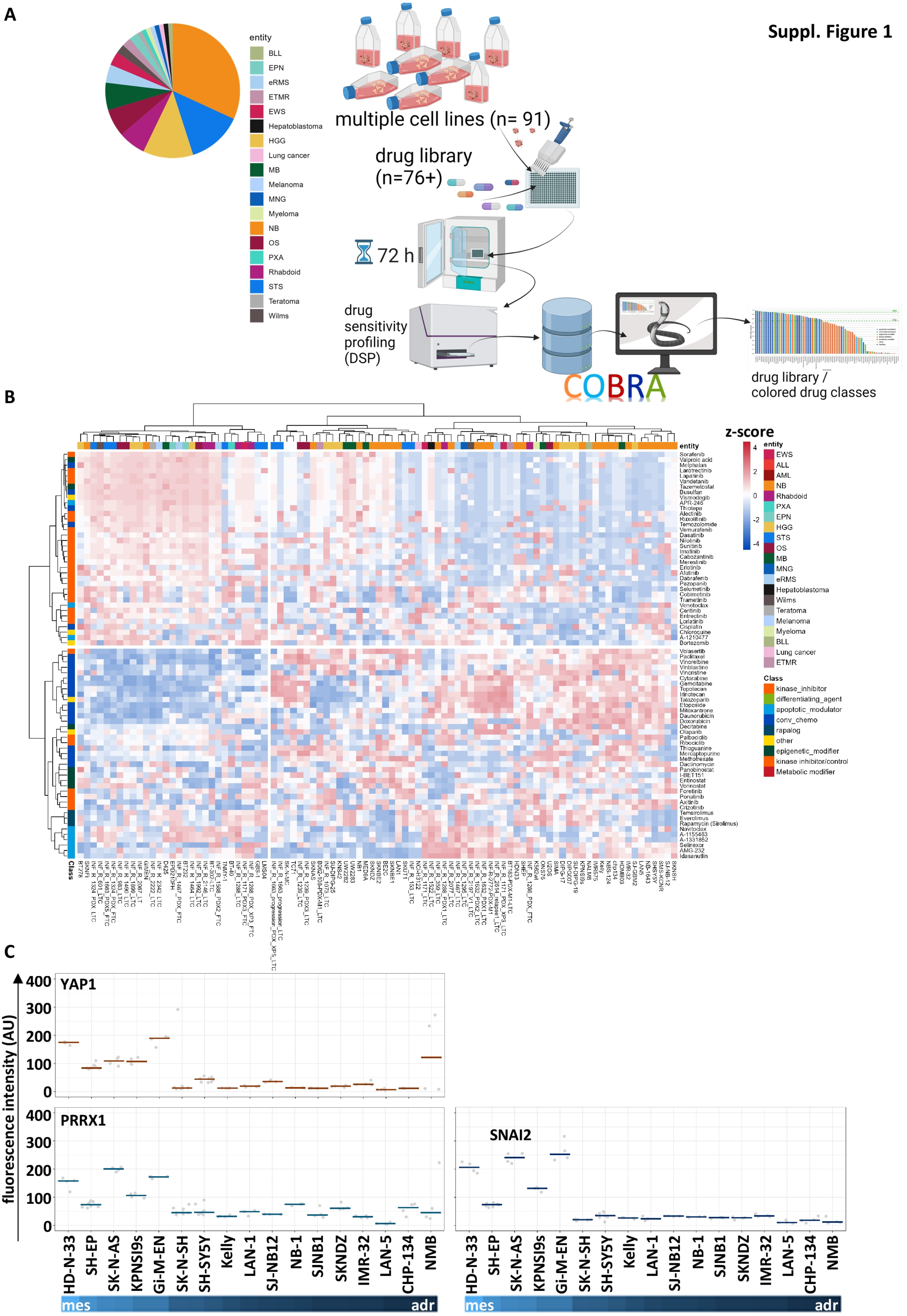

Suppl. Figure 2

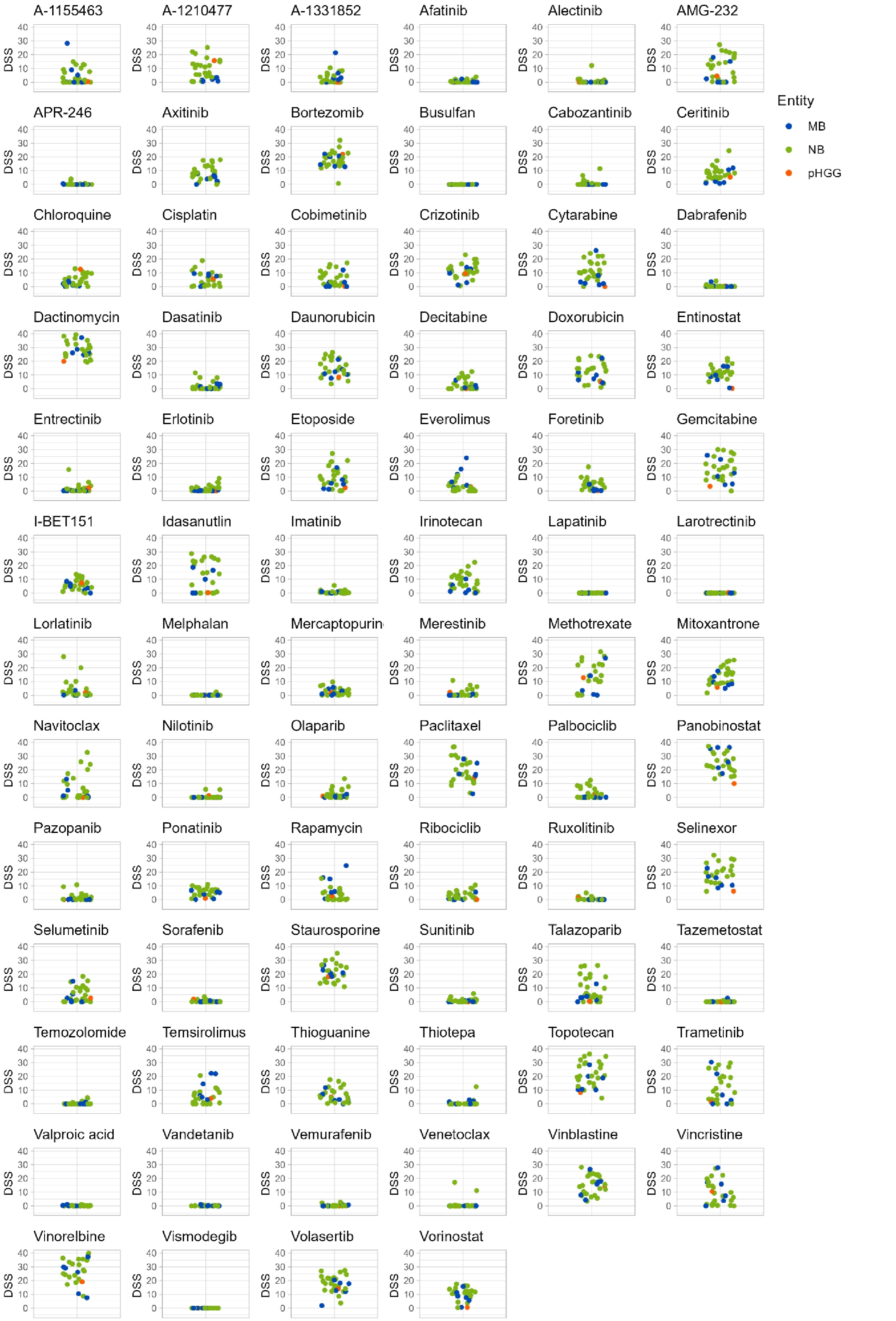

Suppl. Figure 3

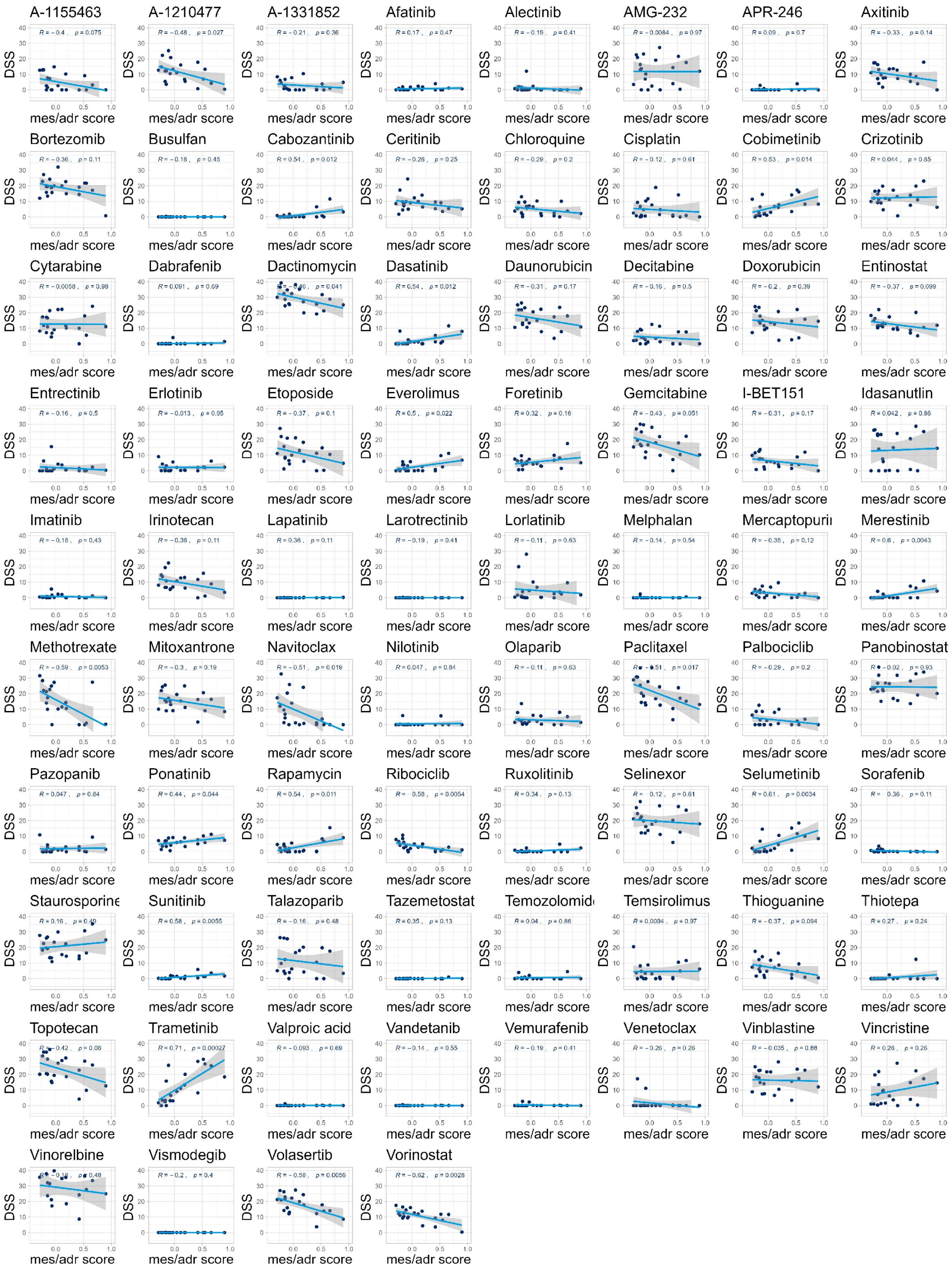

Suppl. Figure 4

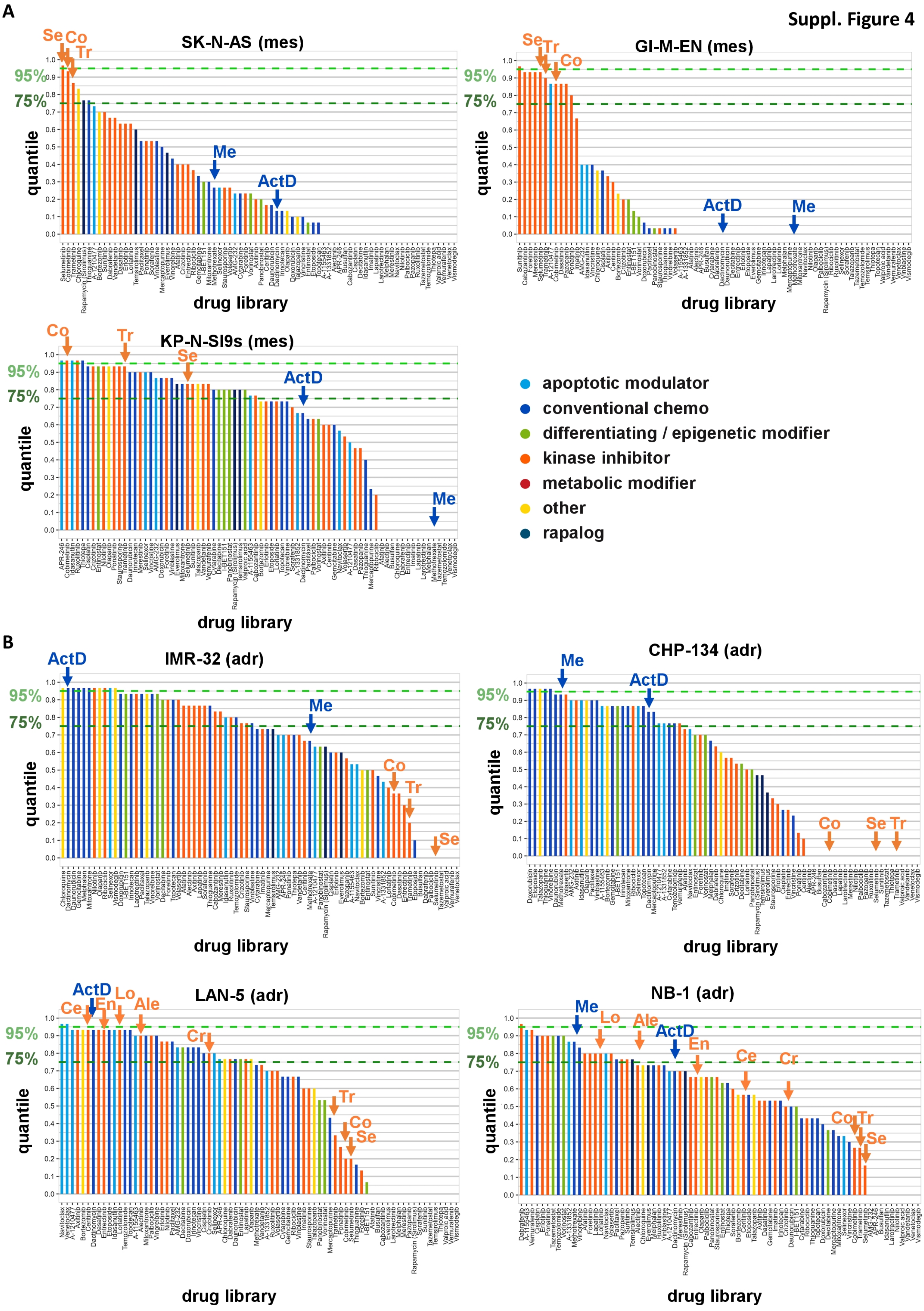

Suppl. Figure 5

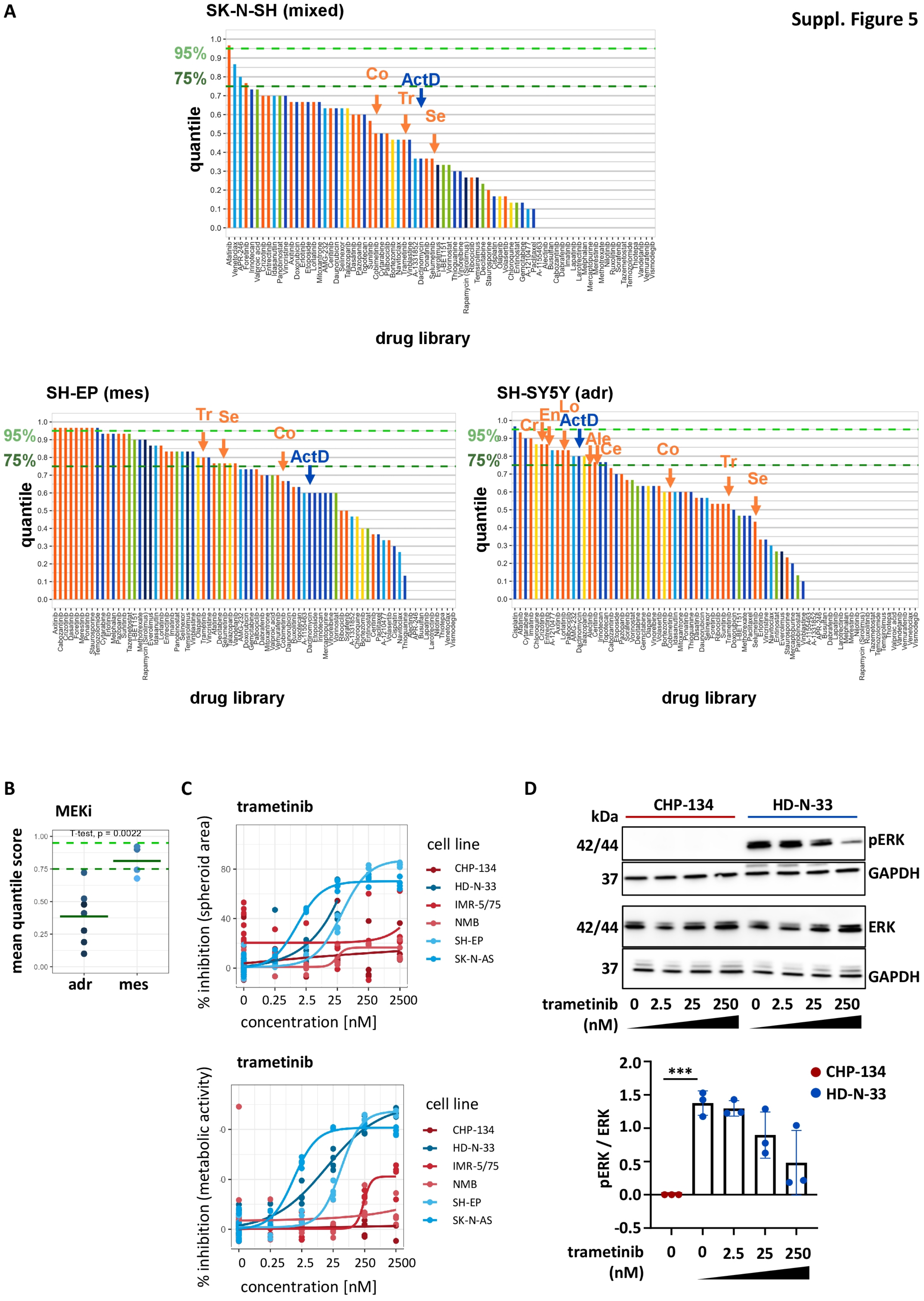

Suppl. Fig. 6

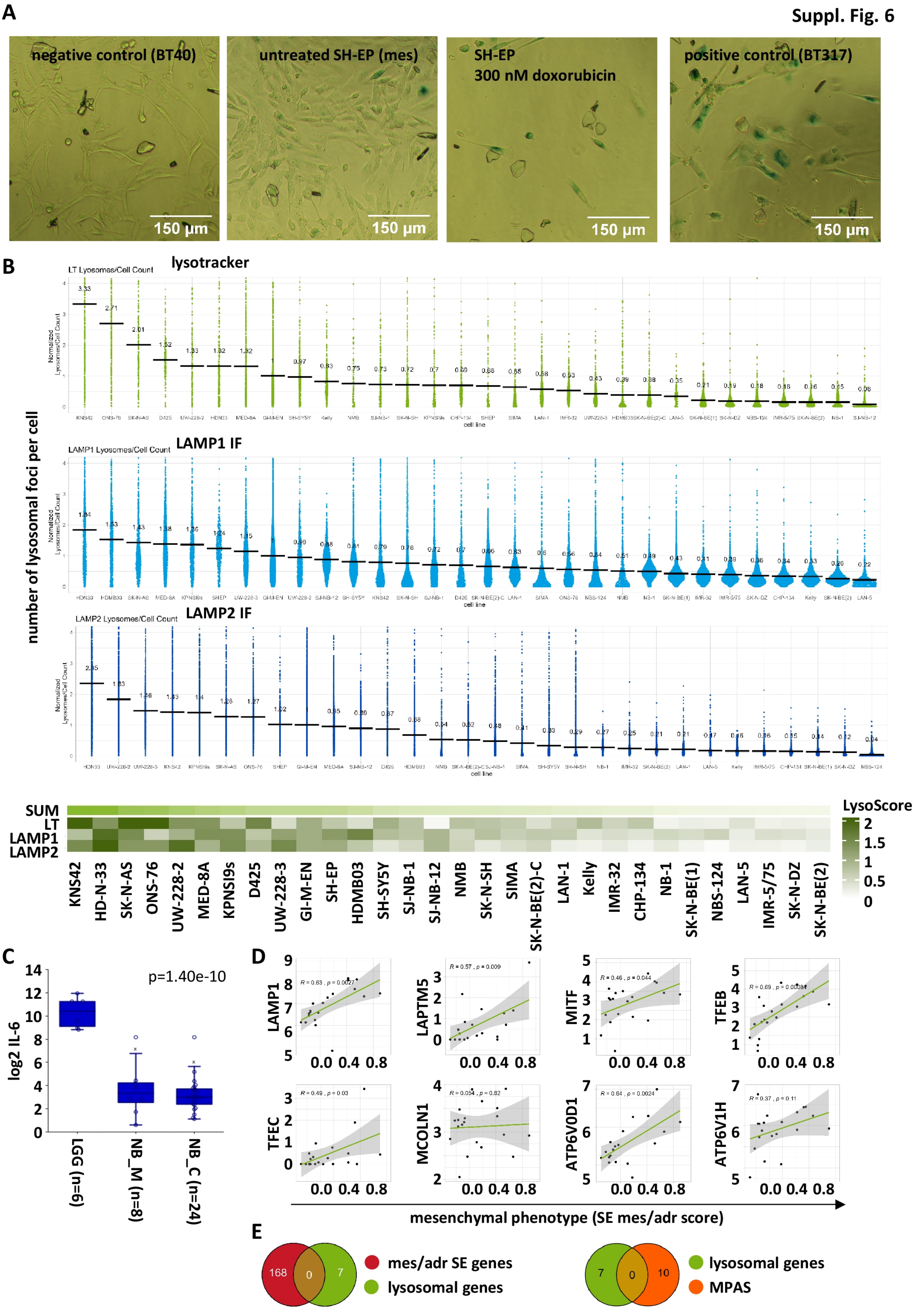
A
negative control (BT40)
untreated SH-EP (mes)
SH-EP
300 nM doxorubicin
positive control (BT317)
150 µm
B
lysotracker
LT Lysosomes/Cell Count
LAMP1 IF
LAMP1 Lysosomes/Cell Count
LAMP2 IF
LAMP2 Lysosomes/Cell Count
number of lysosomal foci per cell
Normalized Lysosomes/Cell Count
cell line
SUM
LT
LAMP1
LAMP2
LysoScore
KNS42
HD-N-33
SK-N-AS
ONS-76
UW-228-2
MED-8A
KPNSI9s
D425
UW-228-3
GI-M-EN
SH-EP
HDMB03
SH-SY5Y
SJ-NB-1
SJ-NB-12
NMB
SK-N-SH
SIMA
SK-N-BE(2)-C
LAN-1
Kelly
IMR-32
CHP-134
NB-1
SK-N-BE(1)
NBS-124
LAN-5
IMR-5/75
SK-N-DZ
SK-N-BE(2)
C
log2 IL-6
p=1.40e-10
LGG (n=6)
NB_M (n=8)
NB_C (n=24)
D
LAMP1
LAPTM5
MITF
TFEB
TFEC
MCOLN1
ATP6V0D1
ATP6V1H
R = 0.63 , p = 0.0027
R = 0.57 , p = 0.009
R = 0.46 , p = 0.044
R = 0.69 , p = 0.00084
R = 0.49 , p = 0.03
R = 0.054 , p = 0.82
R = 0.64 , p = 0.0024
R = 0.37 , p = 0.11
mesenchymal phenotype (SE mes/adr score)
E
168
0
7
mes/adr SE genes
lysosomal genes
10
MPAS

Suppl. Fig. 7

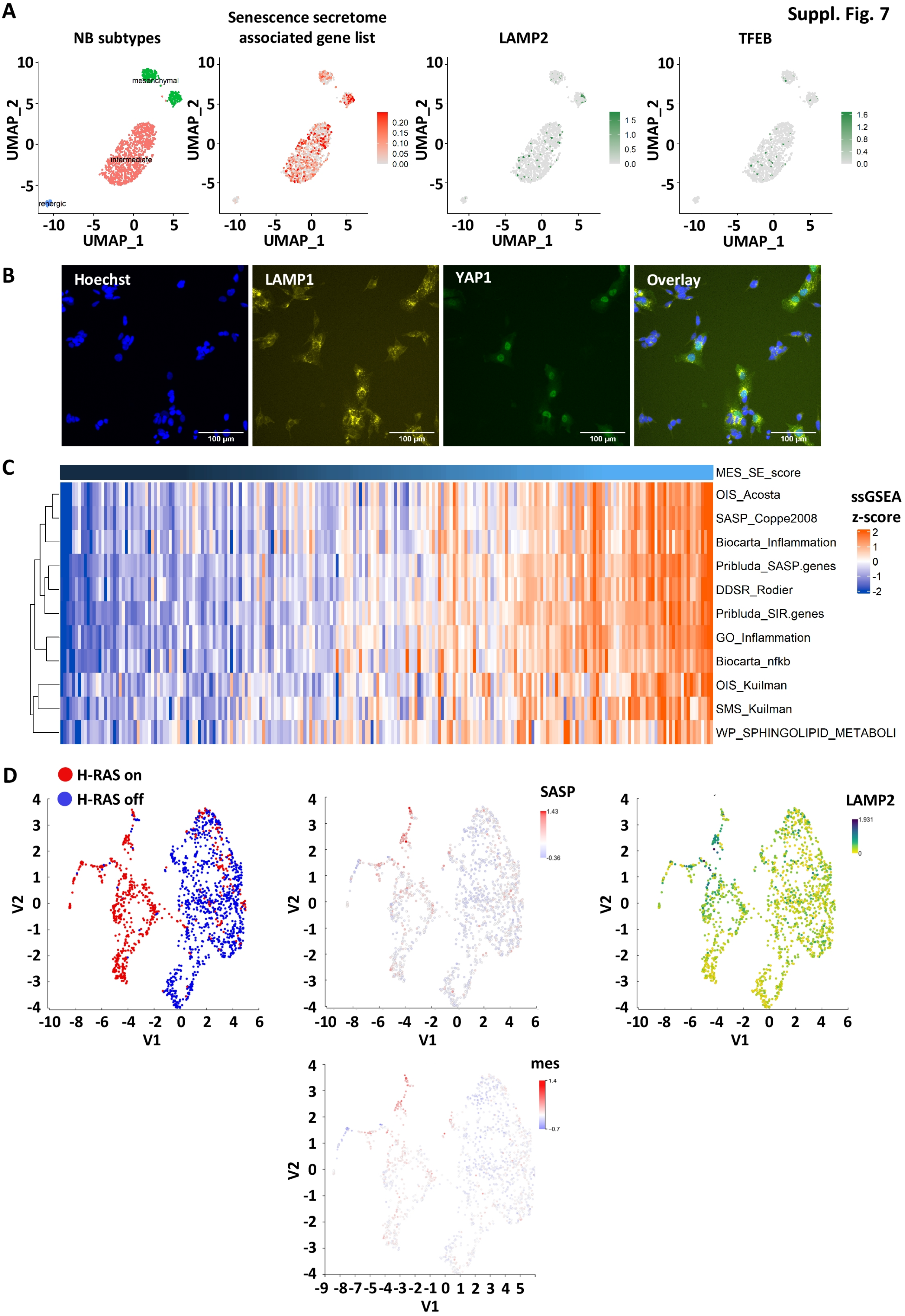
A
NB subtypes
mesenchymal
intermediate
renergic
Senescence secretome associated gene list
LAMP2
TFEB
UMAP_1
UMAP_2
B
Hoechst
LAMP1
YAP1
Overlay
100 µm
C
MES_SE_score
OIS_Acosta
SASP_Coppe2008
Biocarta_Inflammation
Pribluda_SASP.genes
DDSR_Rodier
Pribluda_SIR.genes
GO_Inflammation
Biocarta_nfkb
OIS_Kuilman
SMS_Kuilman
WP_SPHINGOLIPID_METABOLI
ssGSEA z-score
D
H-RAS on
H-RAS off
SASP
LAMP2
mes
V1
V2

Suppl. Fig. 8

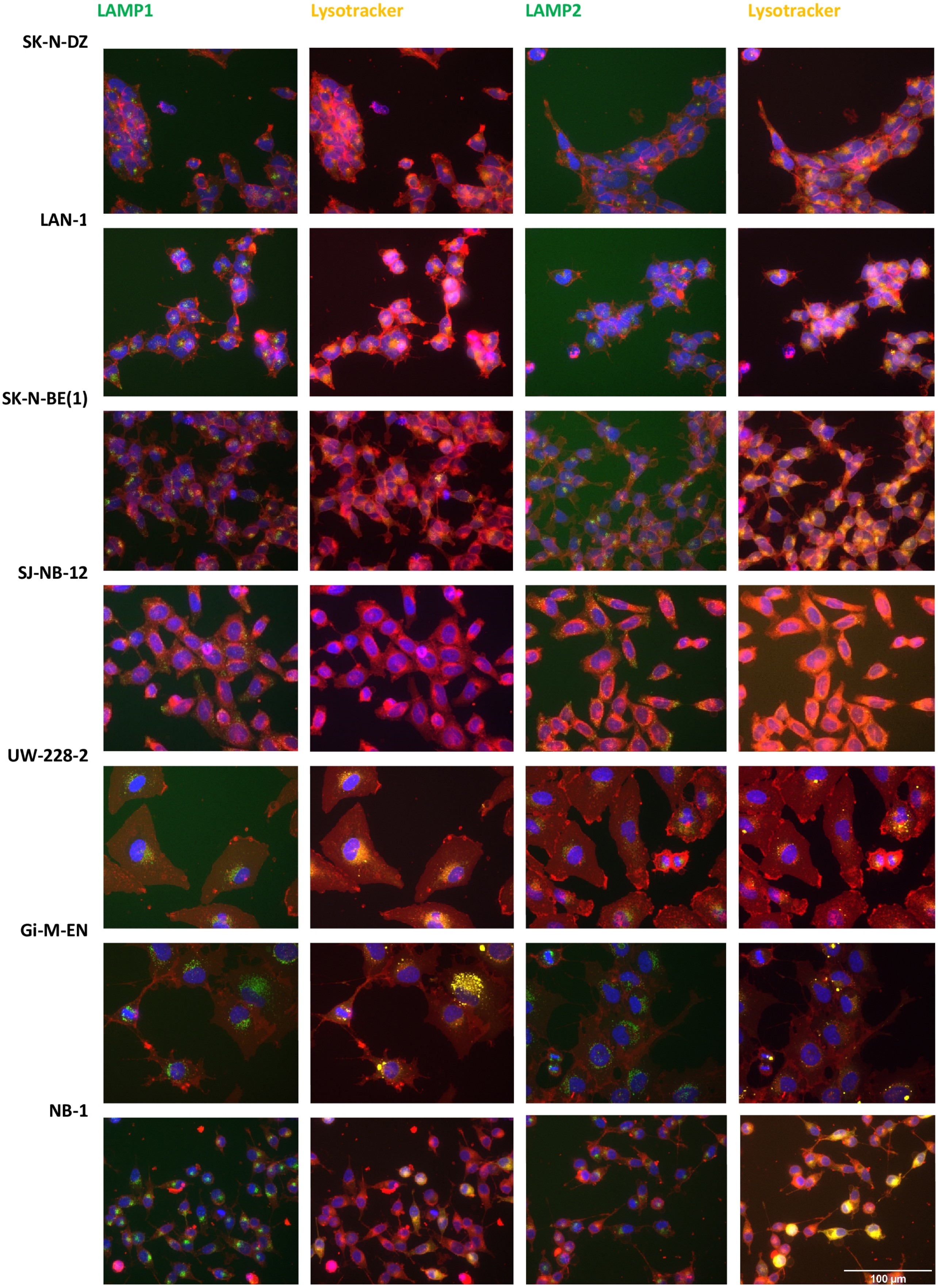
LAMP1
Lysotracker
LAMP2
Lysotracker
SK-N-DZ
LAN-1
SK-N-BE(1)
SJ-NB-12
UW-228-2
Gi-M-EN
NB-1
100 µm

Suppl. Fig. 9

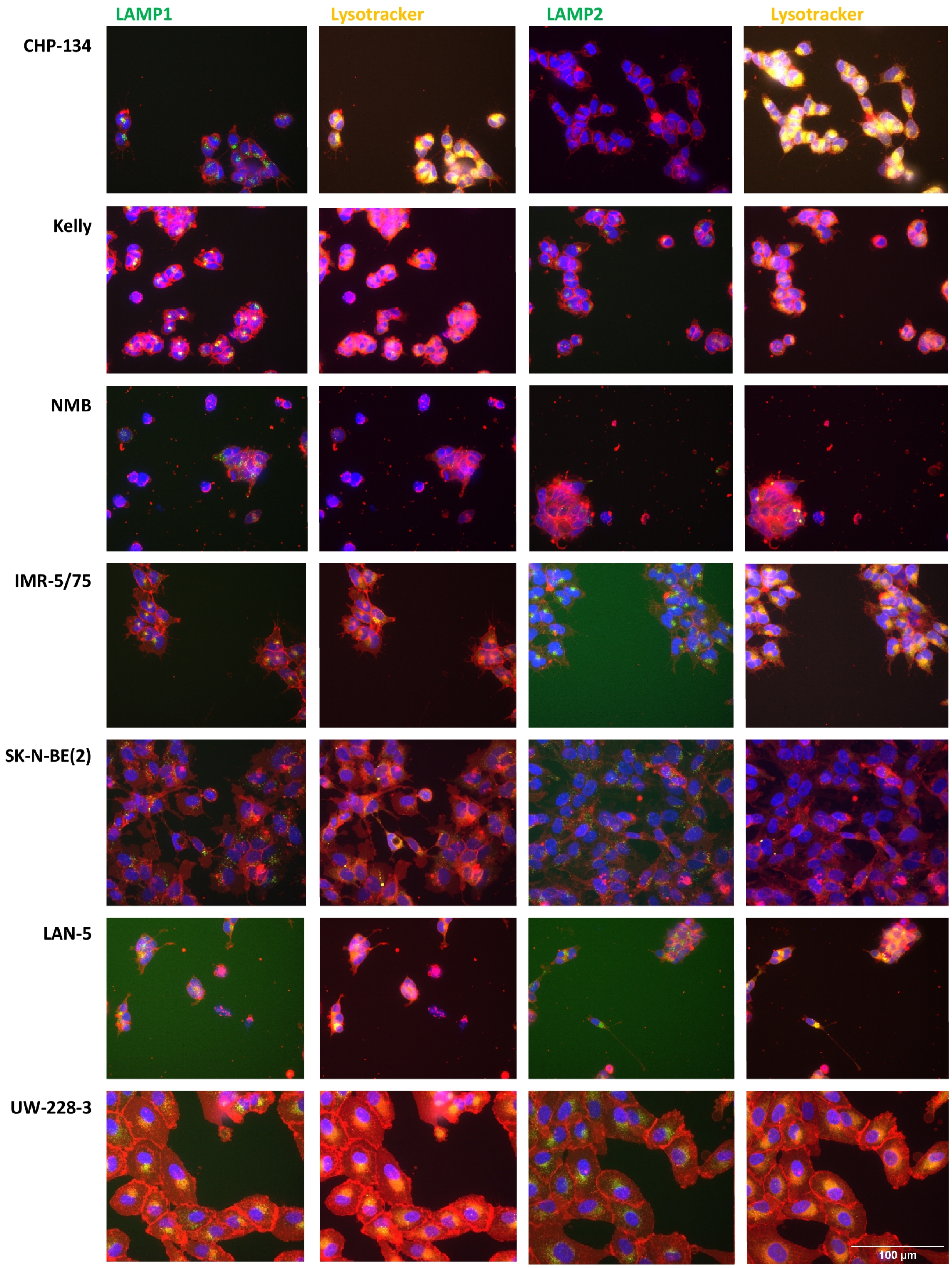
LAMP1
Lysotracker
LAMP2
Lysotracker
CHP-134
Kelly
NMB
IMR-5/75
SK-N-BE(2)
LAN-5
UW-228-3
100 µm

Suppl. Fig. 10

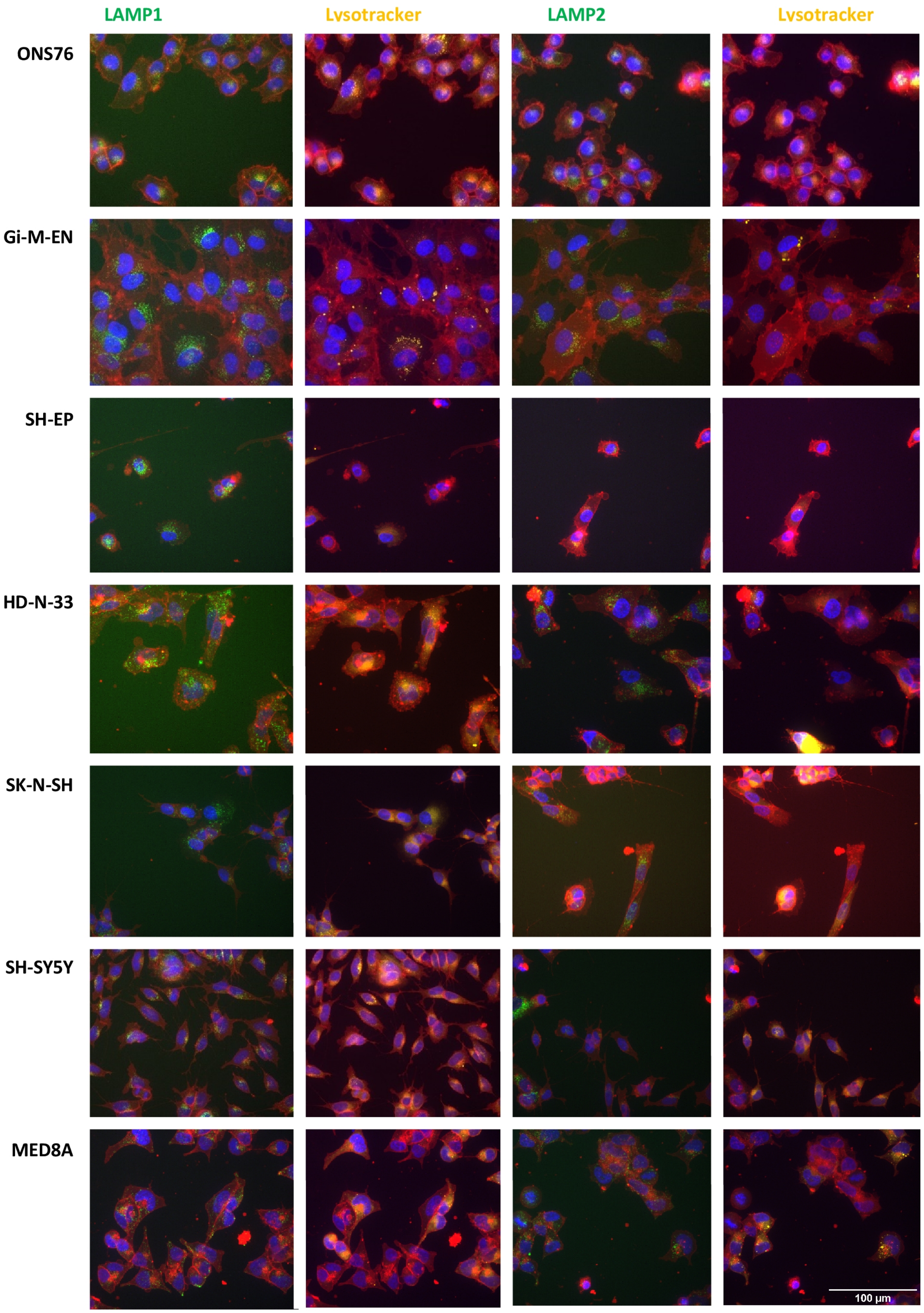
LAMP1
Lysotracker
LAMP2
Lysotracker
ONS76
Gi-M-EN
SH-EP
HD-N-33
SK-N-SH
SH-SY5Y
MED8A
100 µm

Suppl. Fig. 11

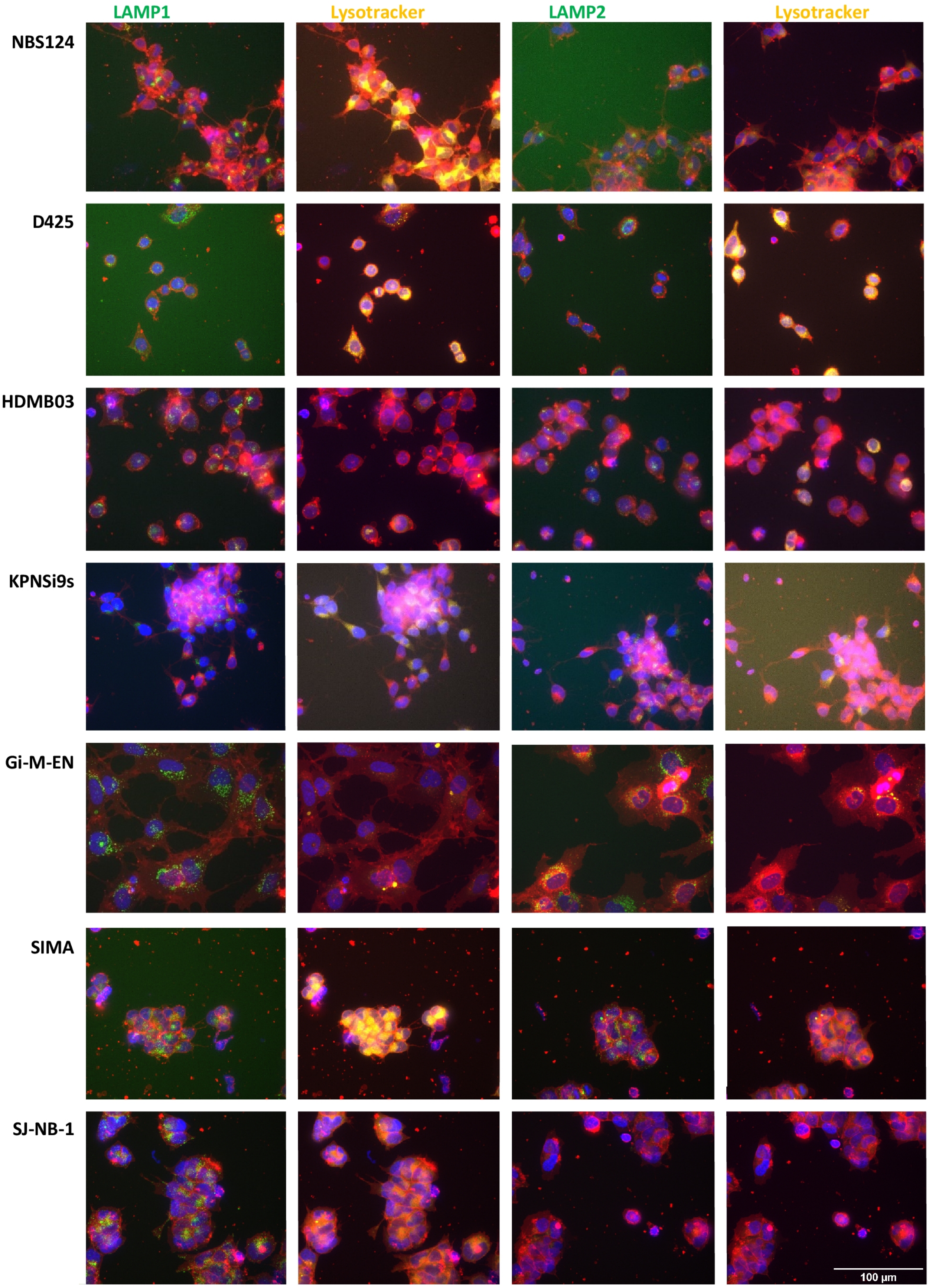
LAMP1
Lysotracker
LAMP2
Lysotracker
NBS124
D425
HDMB03
KPNSi9s
Gi-M-EN
SIMA
SJ-NB-1
100 µm

Suppl. Fig. 12

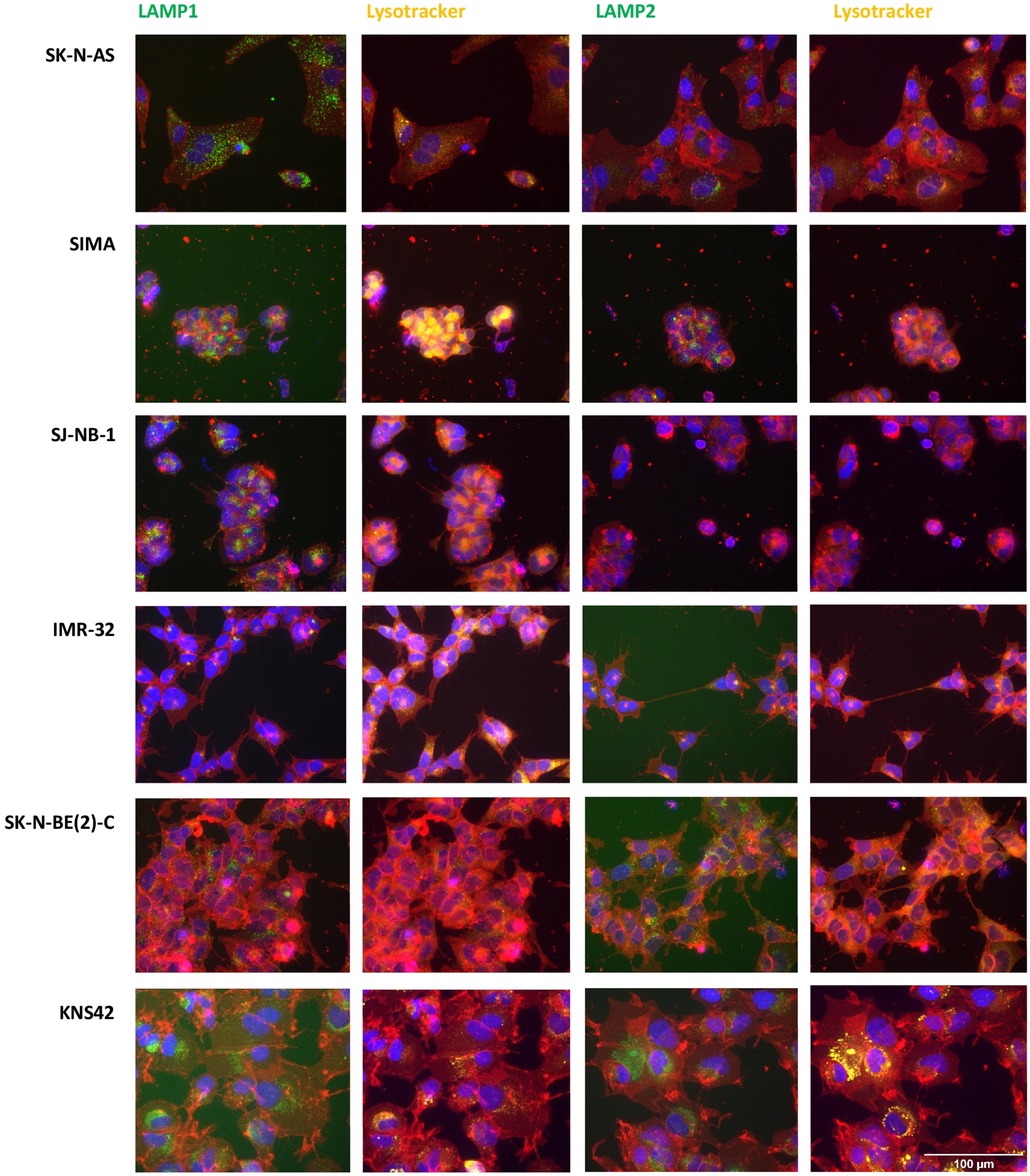
LAMP1
Lysotracker
LAMP2
Lysotracker
SK-N-AS
SIMA
SJ-NB-1
IMR-32
SK-N-BE(2)-C
KNS42
100 µm

Suppl. Figure 13

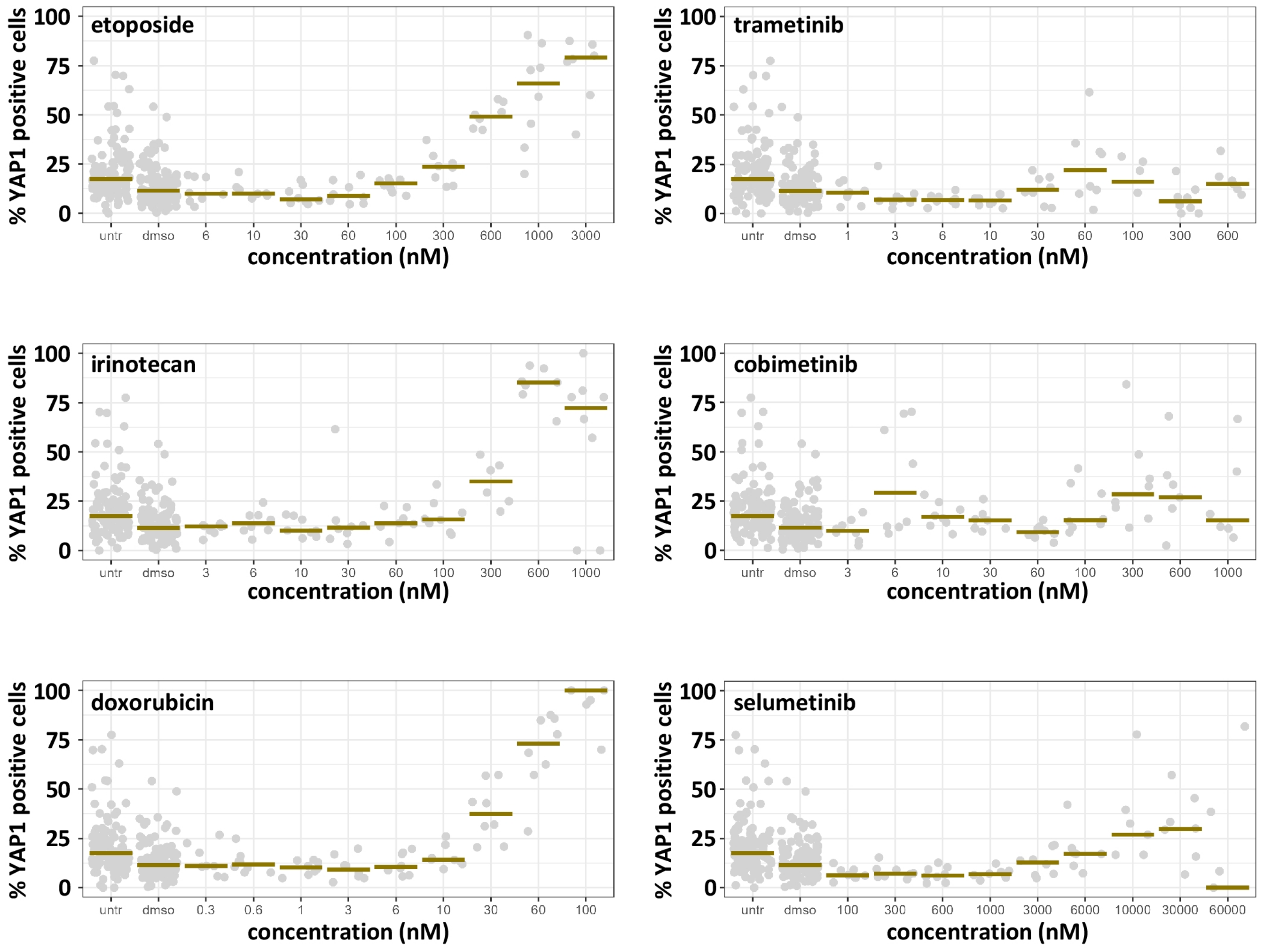

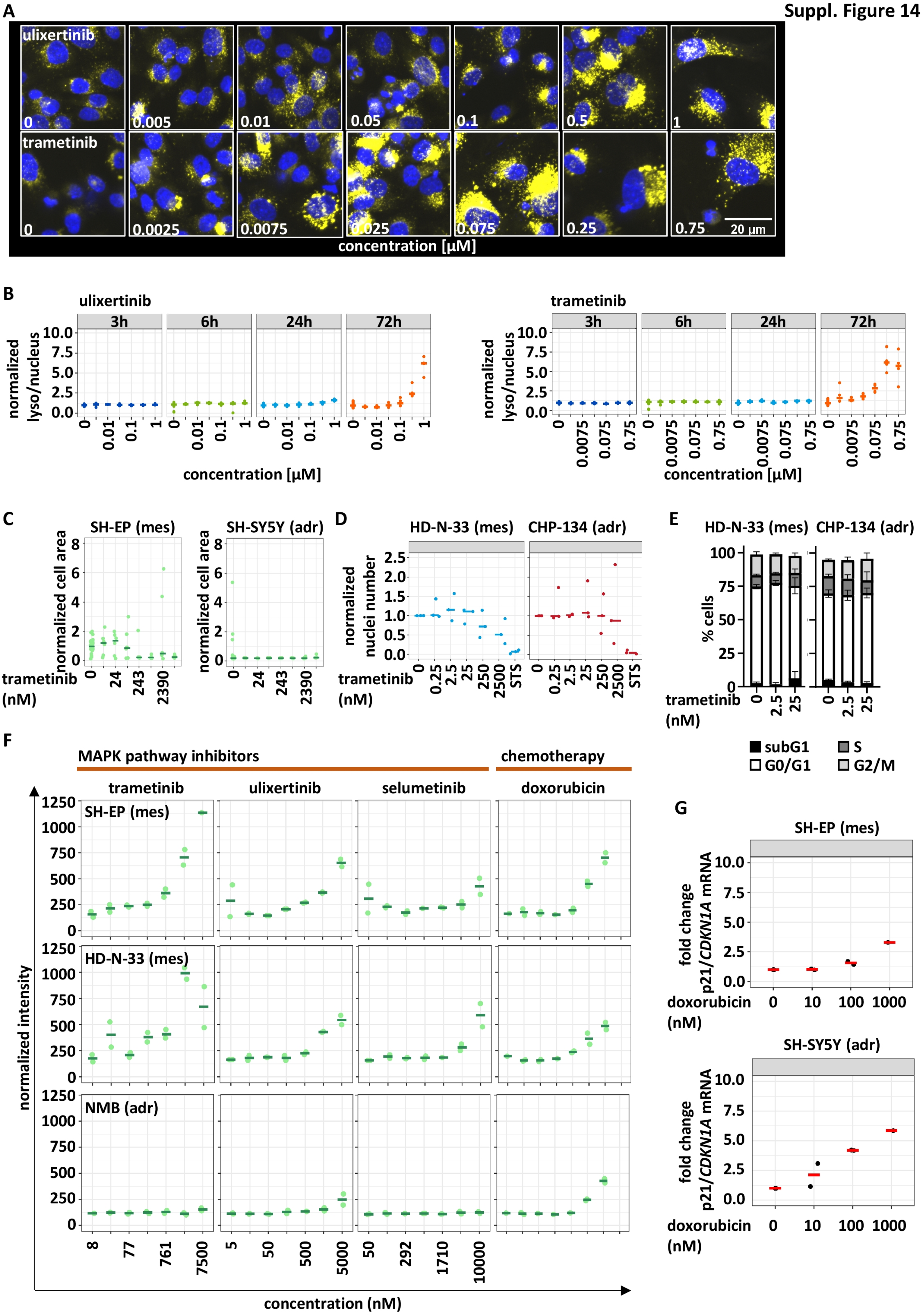
Suppl. Figure 14
A
ulixertinib
0
0.005
0.01
0.05
0.1
0.5
1
trametinib
0
0.0025
0.0075
0.025
0.075
0.25
0.75
20 µm
concentration [µM]
B
ulixertinib
trametinib
3h
6h
24h
72h
normalized lyso/nucleus
concentration [µM]
C
SH-EP (mes)
SH-SY5Y (adr)
normalized cell area
trametinib (nM)
D
HD-N-33 (mes)
CHP-134 (adr)
normalized nuclei number
STS
E
% cells
subG1
S
G0/G1
G2/M
F
MAPK pathway inhibitors
chemotherapy
trametinib
ulixertinib
selumetinib
doxorubicin
SH-EP (mes)
HD-N-33 (mes)
NMB (adr)
normalized intensity
concentration (nM)
G
SH-EP (mes)
SH-SY5Y (adr)
fold change p21/CDKN1A mRNA
doxorubicin (nM)

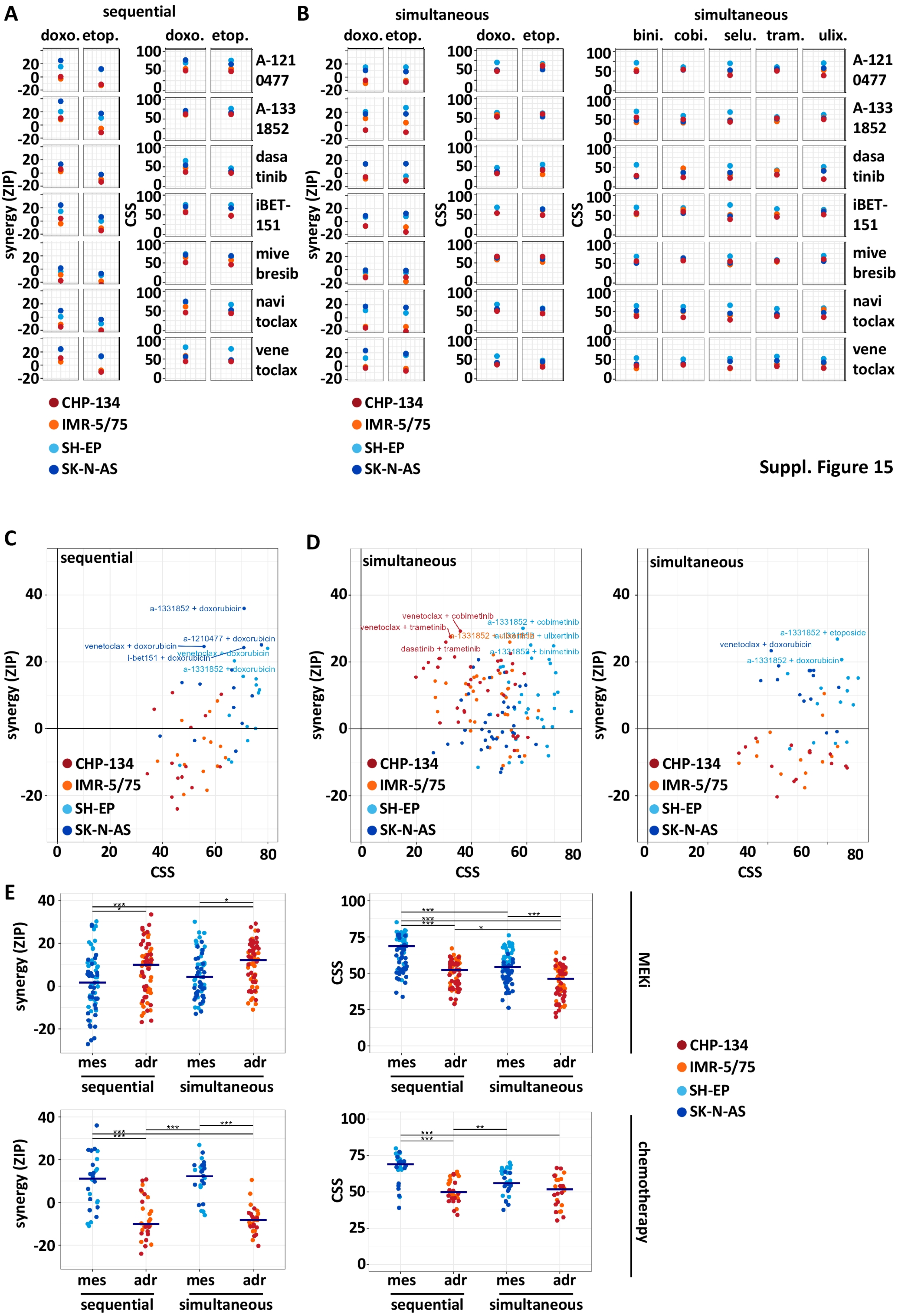

Suppl. Figure 15

Suppl. Figure 16

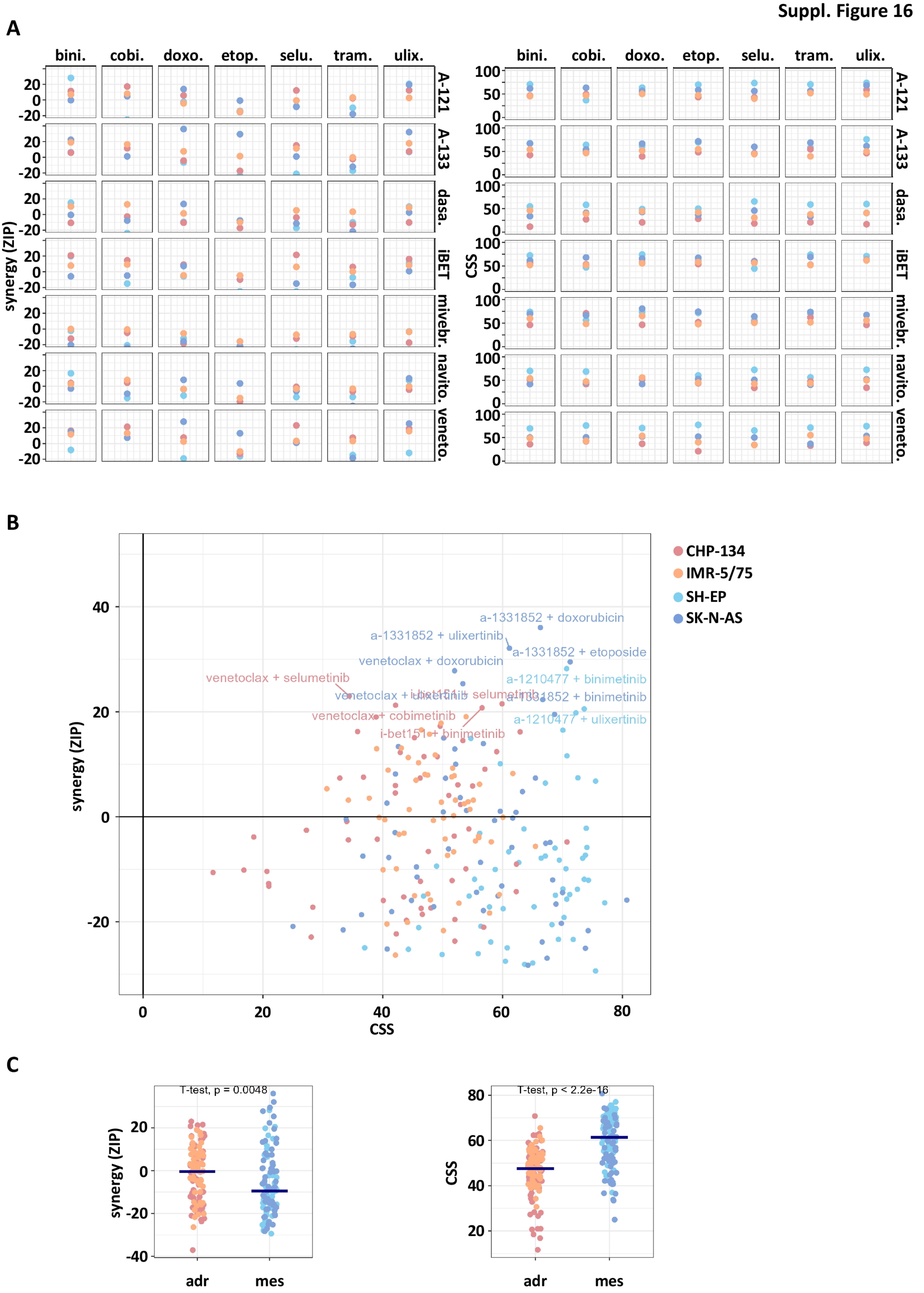

Suppl. Figure 17

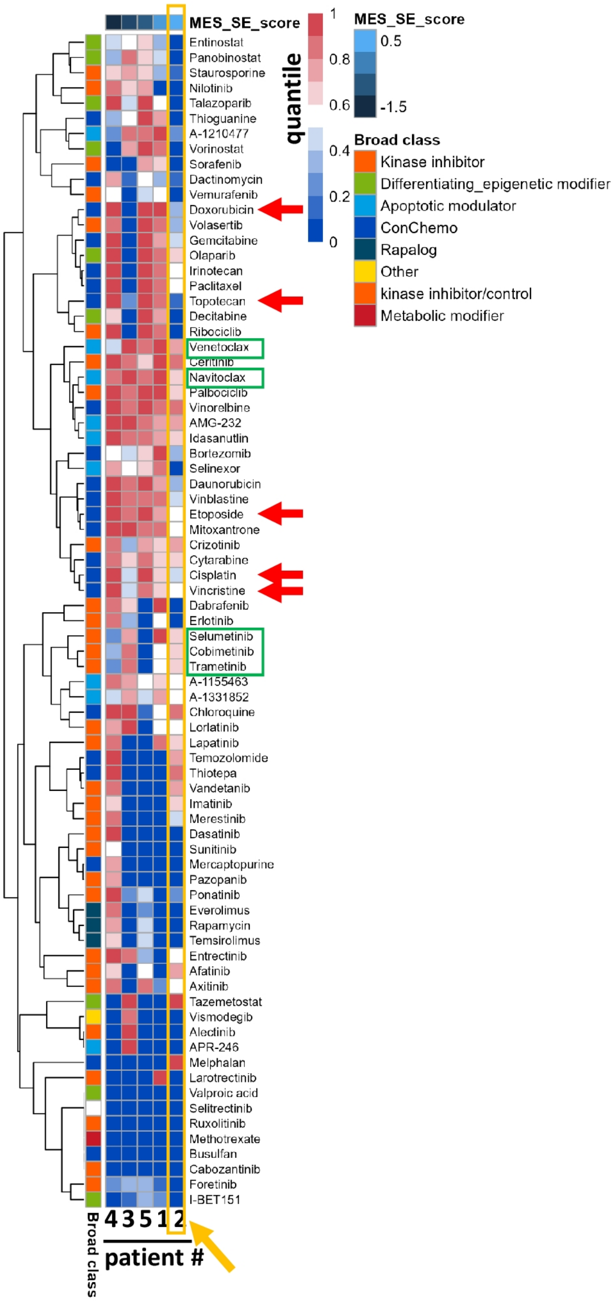

cells	entity	#RRID
B062-108-PDX-M1_LTC	ETMR	ITCCP4 Project
BT183-PDX-M1-LTC	ETMR	Jennifer A.Chan, Dana Faber Institute, Boston, MA, USA
BT232	EPN	ITCCP4 Project
BT278	HGG	INFORM Registry
BT-302-LTC	Rhabdoid	ITCCP4 Project
BT-40	PXA	Peter J Houghton, University of Texas Health Science Center at San Antonio, San Antonio, TX, USA,
chp134	NB	CVCL_1124
D425	MB	CVCL_1275
DIPG007	HGG	CVCL_VU70
DIPG-17	HGG	CVCL_C1MW
EPD210FH	EPN	Brain Tumor Resource lab, Seattle, WA, USA
GBS-1	STS	CVCL_M818
GIMEN	NB	CVCL_1232
HDMB03	MB	CVCL_S506
HDN33	NB	CVCL_W588
IMR-32	NB	CVCL_0346
IMR575	NB	CVCL_M473
INF_R_1073_LTC	HGG	INFORM Registry
INF_R_1171_LTC	EWS	INFORM Registry
INF_R_1171_PDX_XP3_LTC	EWS	INFORM Registry
INF_R_1171_PDX3_FTC	EWS	INFORM Registry
INF_R_1239_LTC	OS	INFORM Registry
INF_R_1239_PDX3_LTC	OS	INFORM Registry
INF_R_1288_LTC	Rhabdoid	INFORM Registry
INF_R_1288_PDX_FTC	Rhabdoid	INFORM Registry
INF_R_1288_PDX_XP3_FTC	Rhabdoid	INFORM Registry
INF_R_1288_PDX1_LTC	Rhabdoid	INFORM Registry
INF_R_1295_LTC	MNG	INFORM Registry
INF_R_1324_PDX_FTC	STS	INFORM Registry
INF_R_1324_PDX_LTC	STS	INFORM Registry
INF_R_1464_LTC	HGG	INFORM Registry
INF_R_1467_LTC	eRMS	INFORM Registry
INF_R_1467_PDX_FTC	eRMS	INFORM Registry
INF_R_1490_LTC	OS	INFORM Registry
INF_R_1522_LTC	Hepatoblastoma	INFORM Registry
INF_R_153_LTC	STS	INFORM Registry
INF_R_1588_PDX2_FTC	eRMS	INFORM Registry
INF_R_1632_PDX1_LTC	NB	INFORM Registry
INF_R_1632_PDX2_LTC	NB	INFORM Registry
INF_R_1663_PDX5_FTC	STS	INFORM Registry
INF_R_1663_progression_LTC	STS	INFORM Registry
INF_R_1663_progression_PDX_XP5_LTC	STS	INFORM Registry
INF_R_1899_LTC	HGG	INFORM Registry

INF_R_1992_LTC	OS	INFORM Registry
INF_R_2067_LT	HGG	INFORM Registry
INF_R_2077_LTC	NB	INFORM Registry
INF_R_2146_LTC	Rhabdoid	INFORM Registry
INF_R_2197_V1_LTC	Wilms	INFORM Registry
INF_R_2222_LTC	Teratoma	INFORM Registry
INF_R_2342_LTC	Melanoma	INFORM Registry
INF_R_2614_relapse1_LTC	eRMS	INFORM Registry
INF_R_359_LTC	NB	INFORM Registry
INF_R_601_LTC	Wilms	INFORM Registry
INF_R_983_LTC	OS	INFORM Registry
INF-R-2772-PDX-M1	NB	INFORM Registry
K562wt	Myeloma	CVCL_0004
Kelly	NB	CVCL_2092
KNS42	HGG	CVCL_0378
KPNSI9s	NB	CVCL_1340
LAN1	NB	CVCL_1827
LAN5	NB	CVCL_0389
LMS04	STS	CVCL_5H99
MED8A	MB	CVCL_M137
NALM6	BLL	CVCL_0092
NB1	NB	CVCL_1440
NB-1643	NB	CVCL_5627
NBS-124	NB	Westermann/Schwab, established at DKFZ
NCI-H3122	Lung cancer	CVCL_5160
ONS76	MB	CVCL_1624
SHEP	NB	CVCL_0524
SHSY5Y	NB	CVCL_0019
SIMA	NB	CVCL_1695
SJ-GBM2	HGG	CVCL_M141
SJ-NB-12	NB	CVCL_1442
SKNAS	NB	CVCL_1700
SK-N-BE(2)-C	NB	CVCL_0529
SKNBE1	NB	CVCL_9898
SKNBE2	NB	CVCL_0528
SKNDZ	NB	CVCL_1701
SKNFi	NB	CVCL_1702
SKNSH	NB	CVCL_0531
SKUT1	STS	CVCL_0533
SMS-KCNR	NB	CVCL_7134
SK-N-MC	EWS	CVCL_0530
SU-DIPG-19	HGG	CVCL_C1MV
SU-DIPG-25	HGG	CVCL_C1N0
TNMY-1	STS	CVCL_M836
TC71	EWS	CVCL_2123
U2OS	OS	CVCL_0042

UW2282	MB	CVCL_0572
UW2283	MB	CVCL_0573

Supplemental table 2: drug library

Substance	Sol-vent	c1 [nM]	c2 [nM]	c3 [nM]	c4 [nM]	c5 [nM]	Company	Cat. No.	Part of metabolic drug screen library
A-1155463	DMSO	10000	1000	100	10	1	ChemieTek	CT-A115	yes
A-1210477	DMSO	50000	5000	500	50	5	Active Biochem	A-9036	yes
A-1331852	DMSO	1000	100	10	1	0,1	ChemieTek	CT-A133	yes
Afatinib	DMSO	1000	100	10	1	0,1	Selleck	S1011	yes
Alectinib	DMSO	1000	100	10	1	0,1	ChemieTek	CT-CH542	yes
Alpelisib	DMSO	10000	1000	100	10	1	Medchem Express	HY-15244	no
AMG-232	DMSO	10000	1000	100	10	1	ChemieTek	CT-AMG232	yes
APR-246	DMSO	10000	1000	100	10	1	Tocris Biosciences	3710	yes
APR-246 2nd concentration range	DMSO	100000	10000	1000	100	10	Tocris Biosciences	3710	yes
Axitinib	DMSO	10000	1000	100	10	1	LC Labora-tories	A-1107	yes
Bortezomib	DMSO	1000	100	10	1	0,1	ChemieTek	CT-BZ001	yes
Busulfan	DMSO	10000	1000	100	10	1	Sigma-Aldrich	B2635	yes
Cabozan-tinib	DMSO	1000	100	10	1	0,1	ChemieTek	CT-XL184	yes
Carboplatin	AQ	10000	1000	100	10	1	Hospital pharmacy Heidelberg	Cay13 112-25	no
CCNU (Lomus-tine)	DMSO	10000	1000	100	10	1	Medchem Express	HY-13669	no

Ceritinib	DMSO	2500	250	25	2,5	0,25	Selleck	S7083	yes
Chloro-quine	AQ	100000	10000	100 0	100	10	Sigma-Aldrich	C6628	yes
Cisplatin	AQ	100000	10000	100 0	100	10	Hospital pharmacy Heidelberg	-	yes
Cobime-tinib	DMSO	1000	100	10	1	0,1	Medchem Express	HY-13064	yes
Copanlisib	AQ	1000	100	10	1	0,1	Medchem Express	HY-15346	no
Crizotinib	DMSO	10000	1000	100	10	1	Selleck	S1068-5	yes
Cytarabine	DMSO	10000	1000	100	10	1	Medchem Express	HY-13605	yes
Dabrafenib	DMSO	2500	250	25	2,5	0,25	ChemieTek	CT-DABR	yes
Dactino-mycin	DMSO	1000	100	10	1	0,1	Medchem Express	HY-17559	yes
Dasatinib	DMSO	1000	100	10	1	0,1	LC Labora-tories	D-3307	yes
Dauno-rubicin	DMSO	1000	100	10	1	0,1	Medchem Express	HY-13062	yes
Decitabine	DMSO	10000	1000	100	10	1	Selleck	S1200	yes
Doxo-rubicin	DMSO	1000	100	10	1	0,1	Sigma-Aldrich	D1515	yes
Entinostat	DMSO	10000	1000	100	10	1	ChemieTek	CT-MS275	yes
Entrectinib	DMSO	1000	100	10	1	0,1	Medchem Express	HY-12678	yes
Erdafitinib	DMSO	10000	1000	100	10	1	Medchem Express	HY-18708	no

Erlotinib	DMSO	10000	1000	100	10	1	Medchem Express	HY-50896	yes
Etoposide	DMSO	10000	1000	100	10	1	Medchem Express	HY-13629	yes
Everolimus	DMSO	100	10	1	0,1	0,01	LC Laboratories	E-4040	yes
Foretinib	DMSO	1000	100	10	1	0,1	Selleck	S1111	yes
Gemcita-bine	DMSO	1000	100	10	1	0,1	Medchem Express	95058-81-4	yes
I-BET151	DMSO	10000	1000	100	10	1	ChemieTek	CT-BET151	yes
Idasanutlin	DMSO	10000	1000	100	10	1	Medchem Express	HY-15676	yes
Imatinib	DMSO	10000	1000	100	10	1	Medchem Express	HY-50946	yes
Irinotecan	DMSO	10000	1000	100	10	1	LC Labora-tories	I-4122	yes
Isotretionin	DMSO	10000	1000	100	10	1	Hölzel	TMO-T1611	no
Lapatinib	DMSO	1000	100	10	1	0,1	LC Labora-tories	L-4804	yes
Larotrec-tinib	DMSO	1000	100	10	1	0,1	Medchem Express	HY-12866	yes
Lorlatinib	DMSO	10000	1000	100	10	1	Medchem Express	HY-12215	yes
Melphalan	AQ	10000	1000	100	10	1	Sigma-Aldrich	M2011	yes
Mercapto-purine	DMSO	10000	1000	100	10	1	Medchem Express	HY-13677	yes
Merestinib	DMSO	1000	100	10	1	0,1	Medchem Express	HY-15514A	yes
Metho-threxate	DMSO	5000	500	50	5	0,5	Selleck	S1210	yes
Mitoxan-trone	DMSO	1000	100	10	1	0,1	Medchem Express	HY-13502A	yes
Navitoclax	DMSO	10000	1000	100	10	1	Medchem Express	HY-10087	yes

Nilotinib	DMSO	10000	1000	100	10	1	LC Labora-tories	N-8207	yes
Olaparib	DMSO	10000	1000	100	10	1	LC Labora-tories	O-9201	yes
ONC201	DMSO	10000	1000	100	10	1	Selleck	S7963	no
Paclitaxel	DMSO	1000	100	10	1	0,1	Medchem Express	HY-B0015	yes
Palbociclib	AQ	10000	1000	100	10	1	Selleck	S1116-10	yes
Panobino-stat	DMSO	1000	100	10	1	0,1	LC Laboratorie s	P-3703	yes
Pazopanib	DMSO	10000	1000	100	10	1	LC Laboratorie s	P-6706	yes
Ponatinib	DMSO	1000	100	10	1	0,1	Selleck	S1490	yes
Pralsetinib	DMSO	10000	1000	100	10	1	BIOZOL	TMO-TQ027 7	no
Rapamycin	DMSO	100	10	1	0,1	0,01	LC Labora-tories	R-5000	yes
Ribociclib	DMSO	10000	1000	100	10	1	Selleck	S7440	yes
Romidepsin	DMSO	1000	100	10	1	0,1	Medchem Express	HY-15149	no
Ruxolitinib	DMSO	10000	1000	100	10	1	ChemieTek	CT-INCB-2	yes
Selinexor	DMSO	10000	1000	100	10	1	Selleck	S7252	yes
Selume-tinib	DMSO	10000	1000	100	10	1	Medchem Express	HY-50706	yes
SN-38	DMSO	100	10	1	0,1	0,01	Medchem Express	Hy-13704	no
Sorafenib p-Toluene-sulfonate Salt	DMSO	1000	100	10	1	0,1	LC Labora-tories	S-8502	yes
Sunitinib	DMSO	1000	100	10	1	0,1	LC Labora-tories	S-8803	yes
Talazoparib	DMSO	1000	100	10	1	0,1	Medchem Express	HY-16106	yes
Tazemeto-stat	DMSO	10000	1000	100	10	1	ChemieTek	CT-EPZ43 8	yes

Temozolomide	DMSO	100000	10000	1000	100	10	Selleck	S1237	yes
Temsiroli-mus	DMSO	100	10	1	0,1	0,01	LC Labora-tories	T-8040	yes
Thiogua-nine	DMSO	10000	1000	100	10	1	Medchem Express	HY-13765	yes
Thiotepa	DMSO	50000	5000	500	50	5	Sigma-Aldrich	T6069	yes
Topotecan	DMSO	10000	1000	100	10	1	Medchem Express	HY-13768A	yes
Trametinib	DMSO	2500	250	25	2,5	0,25	ChemieTek	CT-GSK112	yes
Valproic acid	AQ	1000000	100000	10000	1000	100	Sigma-Aldrich	P4543	yes
Vandetanib	DMSO	1000	100	10	1	0,1	LC Labora-tories	V-9402	yes
Vemura-fenib	DMSO	10000	1000	100	10	1	ChemieTek	CT-P4032-2	yes
Venetoclax	DMSO	1000	100	10	1	0,1	ChemieTek	CT-A199-2	yes
Vinblastine	DMSO	1000	100	10	1	0,1	Medchem Express	HY-13780	yes
Vincristine	DMSO	1000	100	10	1	0,1	Selleck	S1241	yes
Vinorelbine	DMSO	10000	1000	100	10	1	Selleck	S4269	yes
Vismodegib	DMSO	10000	1000	100	10	1	LC Labora-tories	V-4050	yes
Volasertib	DMSO	1000	100	10	1	0,1	ChemieTek	CT-BI6727	yes
Vorinostat	DMSO	10000	1000	100	10	1	LC Labora-tories	V-8477	yes

Supplemental Table 3

**Substance**	**Solvent**	**Company**	**Cat. No.**
Amitriptyline	DMSO	Sigma-Aldrich	BP016
Artesunate	DMSO	Selleckchem	S2265
Bafilomycin A1	DMSO	Selleckchem	S1413
Benzethonium chloride	DMSO	Selleckchem	S4162
Binimetinib	DMSO	Selleckchem	S7007
Fluoxetine	DMSO	BIOZOL	SEL-S1333
GW4869	DMSO	Medchem Express	HY-19363
LY3214996	DMSO	Selleckchem	S8534
Nortriptyline	DMSO	Sigma-Aldrich	BP269
Pimasertib	DMSO	Selleckchem	S1475
Ravoxertinib	DMSO	Selleckchem	S7554
SCH772984	DMSO	Selleckchem	S7101
Staurosporine	DMSO	Selleckchem	S1421
Ulixertinib	DMSO	Selleckchem	S7854

WB ERK/pERK rep1 **Gel1** **30.1.2025**
CHP134; HDN-33 cells

Treatment DMSO; Trametinib 2,5nM; 25nM; 250nM
NP-40 cell Lysats after 6h
4-20% TGX Gel; blotting fast blot BioRad
Blocking 5%BSA TBS-T

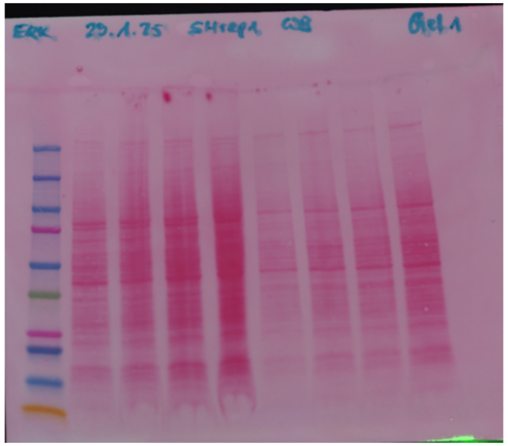

CHP134 | HDN33

DMSO, Trametinib 2,5nM, Trametinib 25nM, Trametinib 250nM, DMSO, Trametinib 2,5nM, Trametinib 25nM, Trametinib 250nM

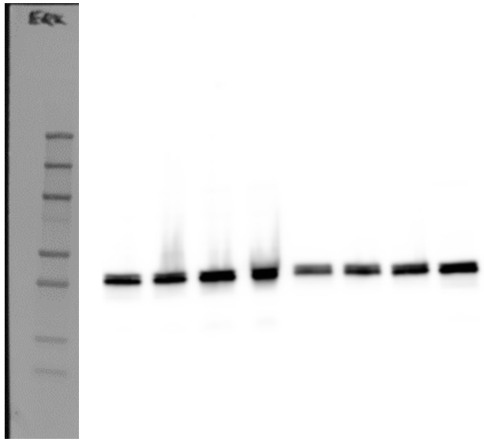

ERK

1 AK: ERK 1:1000 5%BSA TBS-T 1:1000 42/44kDa
2.AK goat anti rabbit in 5%BSA TBS-T 1:20.000

CHP134 | HDN33

DMSO, Trametinib 2,5nM, Trametinib 25nM, Trametinib 250nM, DMSO, Trametinib 2,5nM, Trametinib 25nM, Trametinib 250nM

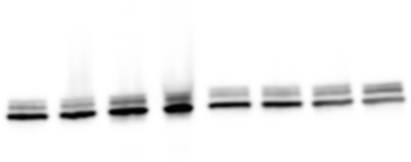

GAPDH

WB ERK/pERK rep1 **Gel2** **30.1.2025**
CHP134; HDN-33 cells

Treatment DMSO; Trametinib 2,5nM; 25nM; 250nM
NP-40 cell Lysats after 6h
4-20% TGX Gel; blotting fast blot BioRad
Blocking TBS-T 5%BSA

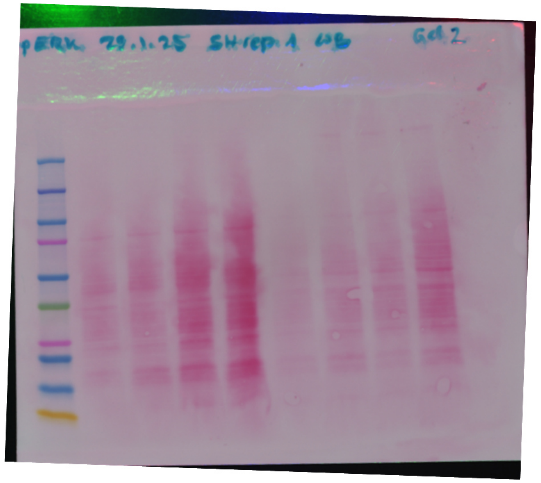

CHP134: DMSO; Trametinib 2,5nM; Trametinib 25nM; Trametinib 250nM

HDN33: DMSO; Trametinib 2,5nM; Trametinib 25nM; Trametinib 250nM

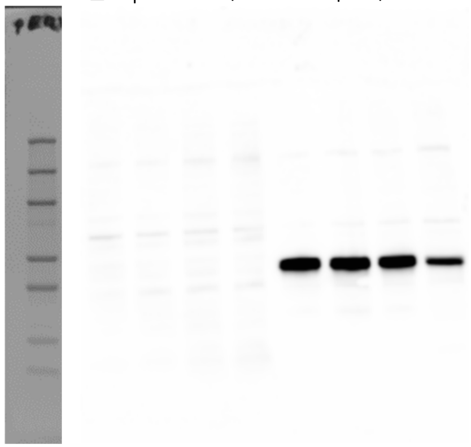

pERK

1 AK: pERK 1:1000 5%BSA TBS-T 1:1000 (42/44kDa)
2.AK goat anti rabbit in 5%BSA TBS-T 1:20.000

WB ERK/pERK rep2 **Gel1** **06.02.2025**
CHP134; HDN-33 cells

Treatment DMSO; Trametinib 2,5nM; 25nM; 250nM
NP-40 cell Lysats after 6h
4-20% TGX Gel; blotting fast blot BioRad
Blocking 5%BSA TBS-T

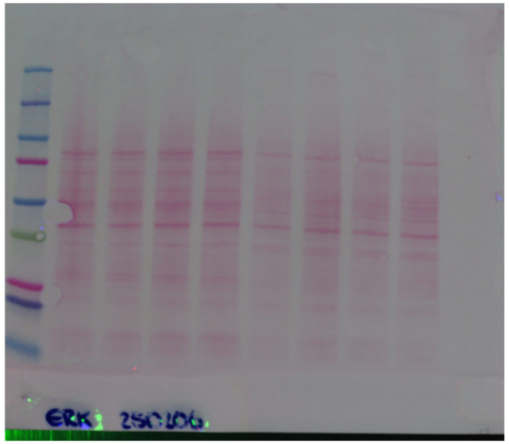

CHP134 | HDN33

DMSO | Trametinib 2,5nM | Trametinib 25nM | Trametinib 250nM | DMSO | Trametinib 2,5nM | Trametinib 25nM | Trametinib 250nM

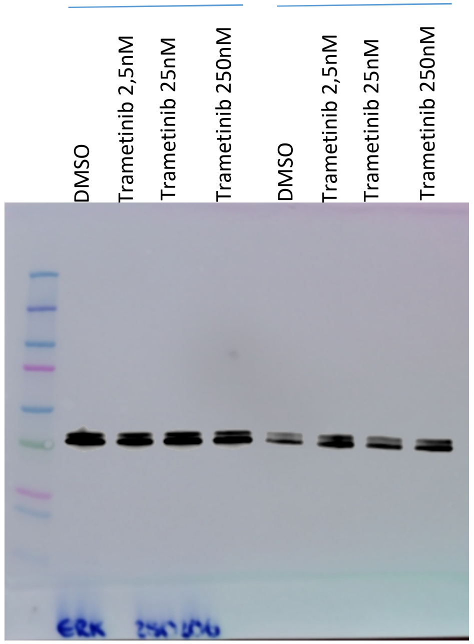

ERK

1 AK: ERK 1:1000 5%BSA TBS-T 1:1000 42/44kDa
2.AK goat anti rabbit in 5%BSA TBS-T 1:20.000

CHP134 | HDN33

DMSO | Trametinib 2,5nM | Trametinib 25nM | Trametinib 250nM | DMSO | Trametinib 2,5nM | Trametinib 25nM | Trametinib 250nM

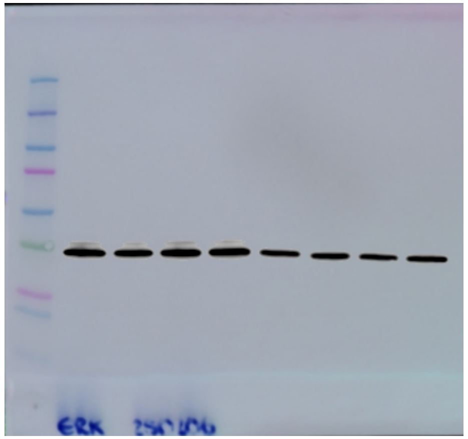

GAPDH

1 AK: GAPDH 1:40.000 5%BSA TBS-T 1:1000 37kDa
2.AK goat anti mouse in 5%BSA TBS-T 1:50.000

WB ERK/pERK rep2 **Gel2** **06.02.2025**

CHP134; HDN-33 cells

Treatment DMSO; Trametinib 2,5nM; 25nM; 250nM
NP-40 cell Lysats after 6h
4-20% TGX Gel; blotting fast blot BioRad
Blocking TBS-T 5%BSA

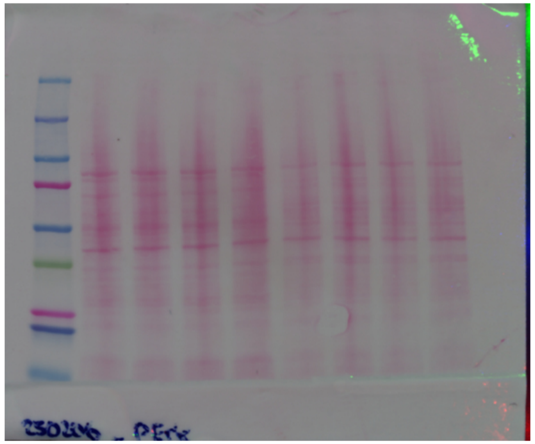

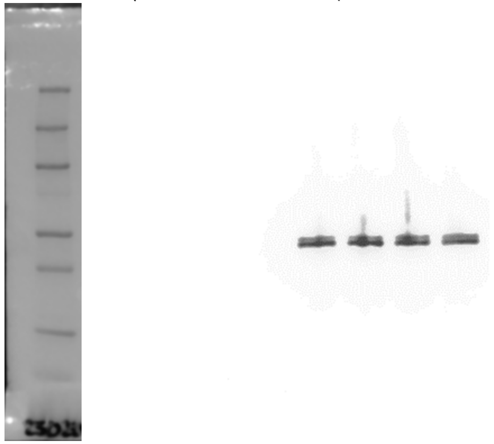

1 AK: pERK 1:1000 5%BSA TBS-T 1:1000 (42/44kDa)
2.AK goat anti rabbit in 5%BSA TBS-T 1:20.000

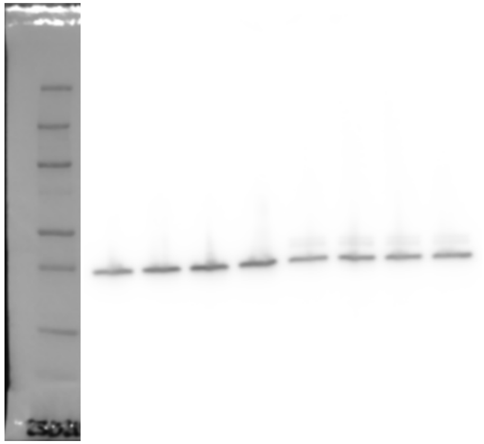

1 AK: GAPDH 1: 40.000 5%BSA TBS-T 1:1000 37kDa
2.AK goat anti mouse in 5%BSA TBS-T 1:50.000

WB ERK rep3 Gel1 06.03.2025

CHP134; HDN-33 cells

Treatment DMSO; Trametinib 2,5nM; 25nM; 250nM
NP-40 cell Lysats after 6h; 10ug Protein
4-20% TGX Gel; blotting fast blot BioRad
Blocking 5%BSA TBS-T

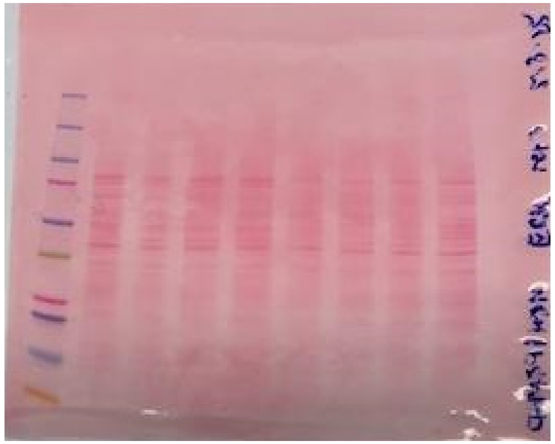

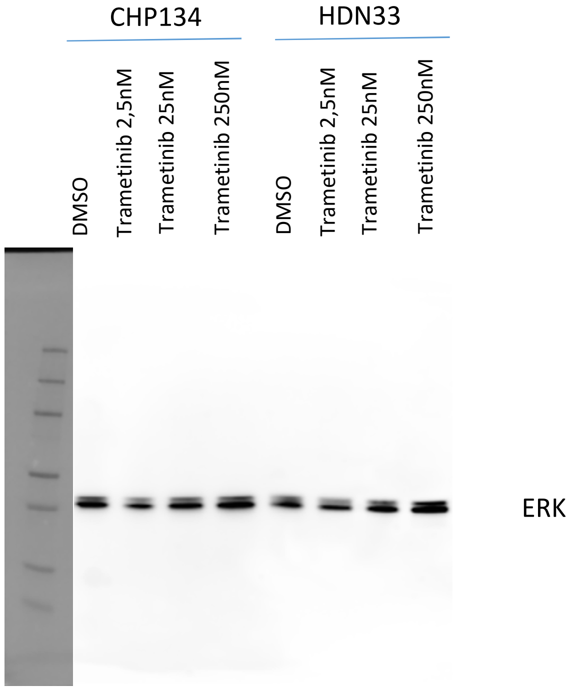

1 AK: ERK 1:1000 5%BSA TBS-T 1:1000 42/44kDa
2.AK goat anti rabbit in 5%BSA TBS-T 1:20.000

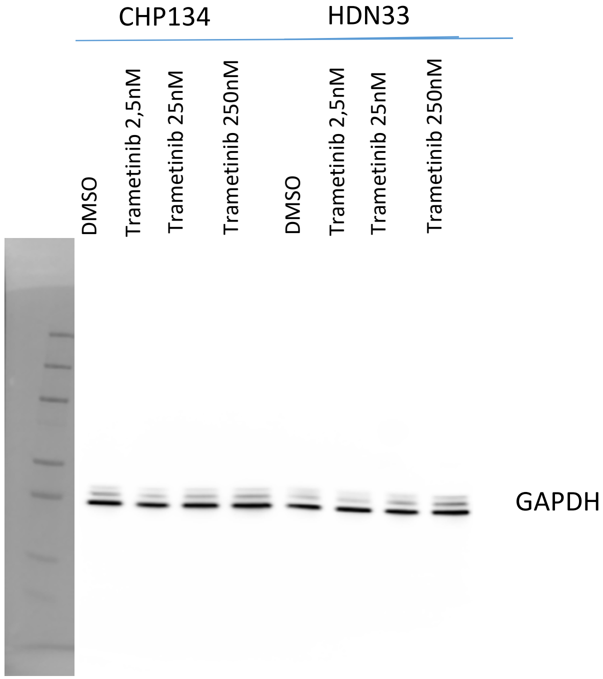

1 AK: GAPDH 1:40.000 5%BSA TBS-T 1:1000 37kDa
2.AK goat anti mouse in 5%BSA TBS-T 1:50.000

WB pERK rep3 **Gel2** **06.03.2025**
CHP134; HDN-33 cells

Treatment DMSO; Trametinib 2,5nM; 25nM; 250nM
NP-40 cell Lysats after 6h; 10ug Protein
4-20% TGX Gel; blotting fast blot BioRad
Blocking TBS-T 5%BSA

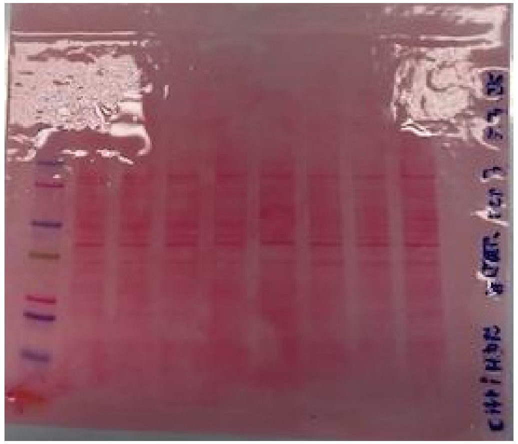

CHP134 HDN33

DMSO | Trametinib 2,5nM | Trametinib 25nM | Trametinib 250nM | DMSO | Trametinib 2,5nM | Trametinib 25nM | Trametinib 250nM

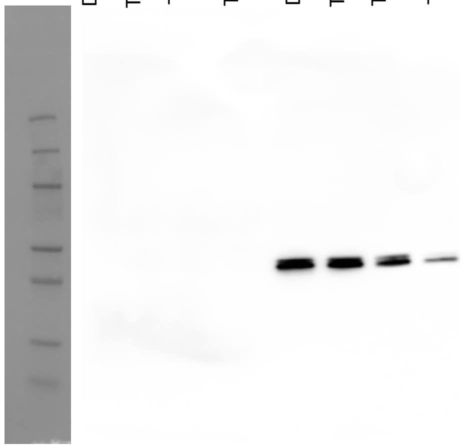

pERK

1 AK: pERK 1:1000 5%BSA TBS-T 1:1000 (42/44kDa)
2.AK goat anti rabbit in 5%BSA TBS-T 1:20.000

CHP134 HDN33

DMSO | Trametinib 2,5nM | Trametinib 25nM | Trametinib 250nM | DMSO | Trametinib 2,5nM | Trametinib 25nM | Trametinib 250nM

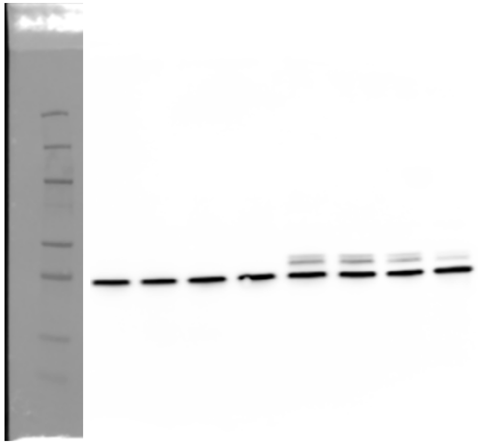

GAPDH

1 AK: GAPDH 1: 40.000 5%BSA TBS-T 1:1000 37kDa
2.AK goat anti mouse in 5%BSA TBS-T 1:50.000
